# Low complexity smart grid security protocol based on elliptic curve cryptography, biometrics and hamming distance

**DOI:** 10.1371/journal.pone.0296781

**Published:** 2024-01-23

**Authors:** Keyan Abdul-Aziz Mutlaq, Vincent Omollo Nyangaresi, Mohd Adib Omar, Zaid Ameen Abduljabbar, Iman Qays Abduljaleel, Junchao Ma, Mustafa A. Al Sibahee

**Affiliations:** 1 School of Computer Sciences, Universiti Sains Malaysia, USM, Gelugor, Penang, Malaysia; 2 IT and Communications Center, University of Basrah, Basrah, Iraq; 3 Department of Computer Science and Software Engineering, Jaramogi Oginga Odinga University of Science & Technology, Bondo, Kenya; 4 Department of Computer Science, College of Education for Pure Sciences, University of Basrah, Basrah, Iraq; 5 Department of Computer Science, College of Computer Science and Information Technology, University of Basrah, Basrah, Iraq; 6 College of Big Data and Internet, Shenzhen Technology University, Shenzhen, China; 7 National Engineering Laboratory for Big Data System Computing Technology, Shenzhen University, Shenzhen, China; 8 Computer Technology Engineering Department, Iraq University College, Basrah, Iraq; University College of Engineering Tindivanam, INDIA

## Abstract

The incorporation of information and communication technologies in the power grids has greatly enhanced efficiency in the management of demand-responses. In addition, smart grids have seen considerable minimization in energy consumption and enhancement in power supply quality. However, the transmission of control and consumption information over open public communication channels renders the transmitted messages vulnerable to numerous security and privacy violations. Although many authentication and key agreement protocols have been developed to counter these issues, the achievement of ideal security and privacy levels at optimal performance still remains an uphill task. In this paper, we leverage on Hamming distance, elliptic curve cryptography, smart cards and biometrics to develop an authentication protocol. It is formally analyzed using the Burrows-Abadi-Needham (BAN) logic, which shows strong mutual authentication and session key negotiation. Its semantic security analysis demonstrates its robustness under all the assumptions of the Dolev-Yao (DY) and Canetti- Krawczyk (CK) threat models. From the performance perspective, it is shown to incur communication, storage and computation complexities compared with other related state of the art protocols.

## 1. Introduction

Electrical grids comprise of networks that perform power generation, transmission as well as distribution. In this environment, there is need for communication and coordination with the power control centers so as to control and monitor the grid. To boost power supply quality, incorporate novel communication technologies, enhance efficient energy distribution and minimize energy consumptions, smart grids have been developed [1–4]. In essence, the smart grid (SG) integrates communication and information technologies with power systems so as to enhance reliability, efficiency and sustainable power management. In so doing, SG can potentially alleviate challenges of traditional grid systems such as delayed demand response (DR), blackouts and inefficiency in energy management. In addition, the SG can offer reliable energy distribution, live monitoring of energy consumption, two-way energy flow, better resource allocations, outages prediction and prevention, ideal real-time balance between energy demand and supply, as well as incorporation of micro energy generators such as solar power into the electricity grid. Authors in [5] point out that the SG’s advanced automation and distributed intelligence can provide fault detection, recovery as well as DR management. A typical SG comprises of smart meters, renewable energy resources, smart appliances, distribution networks [6], power plants and transmission networks. As discussed in [7], the SG facilitates the integration of cleaner energy technologies with energy management. This helps in enhancing efficiency and reliability in the power network. In this environment, the smart meter (SM) offers fine-grained home or enterprise power consumption information.

In spite of the numerous merits of the smart grids, many issues remain unresolved in these networks. For instance, the SG requires the deployment of numerous components for control and monitoring. This calls for the amalgamation of power resources at various levels, such as operating systems, cloud databases, networking and smart systems [8]. The massive flow of information in the distributed SG is exposed to many cyber attacks, which compromise its integrity, confidentiality and availability [9–11]. These attacks can have adverse effects on the consumers as well as the operation of the grid. As explained in [12], the SM is the point of contact with the SG and is the vulnerable link. Therefore, malicious SM endpoints may instigate attacks such as Sybil, false data injection, identity theft and consumption report altering. In addition, Denial of Service (DoS), data tampering, Man-in-the-Middle (MitM), impersonation, phishing, Sybil and spoofing attacks as being serious challenges in smart grids. On the other hand, eavesdropping, insertion, modification, deletion, forgery [13] and interception of exchanged messages have been noted in [14] to be critical issues that need urgent solution. Similarly, SGs have been noted in [15] to be susceptible to impersonation attacks, MitM, replays and session key disclosure. These attacks may effectively lead to the forwarding of erroneous feedback to the control center, and hence inappropriate decisions may be made [16]. It may also cause malicious consumption data to the SM, resulting in consumers being charged for energy that they have not utilized [17].

The transmission of messages between consumers and control centers over insecure wireless communications [18] is the major source of SG vulnerabilities. Since the SG security is firmly tied with the data exchange network, it is critical to enforce Authentication, Authorization and Access Control (AAA) at the device level [19]. As explained in [20], proper device identification and authentication can play central responsibility in the elimination of the above attacks. On the other hand, strong identification, management and authentication of SG devices can potentially aid in the prevention of password breaches, identity theft and impersonation [21]. Owing to the limited computation and communication capability of the SG components such as smart meters, lightweight and secure authentication and key agreement (AKA) schemes are needed in this environment. Although numerous AKA schemes have been developed for this purpose, they have various shortcomings that impede their effective deployments.

### A. Motivation

The smart grids are susceptible to numerous attacks such as false command and data injections due to the message exchanges over the open transmission channels. These attacks can cause the utility centers to make erroneous decisions that may have adverse effects such as blackouts. It is also possible for consumer privacy to be compromised such that private information such as economic status, household occupancy and behavioral patterns are discerned. As such, great efforts have been made to develop security solutions in smart grids using techniques such as blockchain, elliptic curve cryptography, public key infrastructure, asymmetric key cryptography, and physical unclonable function. However, most of these approaches incur extensive computation, storage and communication complexities [22]. This limits their deployment in resource-limited smart grid components such as smart gas meters. In addition, most of the current security schemes have susceptibility to numerous attacks and fail to provide mutual authentication, anonymity, non-traceability and key freshness. There is therefore need for novel AKA protocols that address these challenges.

### B. Research contributions

In this sub-section, the major contributions of the proposed protocol are summarized as follows:

1. A protocol leveraging on elliptic curve, smart cards, Hamming distance and biometrics is developed for privacy and security enhancement in smart grid networks.
2. Random nonces are deployed to uphold the freshness of the exchanged messages. This is shown to be crucial in the prevention of de-synchronization attacks that are common in most of the timestamp-based authentication schemes.
3. Formal security is carried out through the BAN logic to demonstrate that the smart grid network entities successfully validate each other and negotiate session keys for traffic encryption.
4. Extensive semantic analysis is executed, which shows that our protocol is robust under all the assumptions of the Canetti- Krawczyk and Dolev-Yao threat models.
5. Comparative performance evaluation is effected, which shows that our protocol incurs lower computation, storage and communication complexities compared with other related schemes.

### C. Paper organization

The rest of this paper is organized structures as follows: Section 2 discusses the related work, while Section 3 details the system model. Conversely, Section 4 presents the proposed protocol while Section 5 details its security analysis. This is followed by the performance analysis in Section 6, while Section 7 concludes the paper and gives future research directions.

## 2. Related work

Security and privacy issues in smart grids have attracted a lot of attention and research work from the industry and academia. As such, numerous AKA schemes have been proposed to address these challenges [23, 24]. For instance, a pairing based anonymous scheme is developed in [25]. However, this scheme is susceptible to ephemeral secret leakage and impersonation attacks [26]. Based on elliptic curve cryptography (ECC), a self-certified scheme is introduced in [27] for key distribution between the SM and control center. However, this technique is vulnerable to DoS attacks and cannot preserve session key security [28]. Similarly, the authentication approach in [29] is susceptible to session key disclosure and impersonation attacks [30]. To address the problems in [29], a provably secure AKA scheme is developed in [30]. Another important technology that can be used to offer energy management, reliability, privacy and security in distributed smart grid environment is the blockchain [31–33]. As such, a blockchain-based security protocol is presented in [15] to offer response management in smart grids. This approach is demonstrated to achieve mutual authentication, key agreement, resist various attacks and offer demand response integrity. However, the blockchain incurs heavy computation and storage overheads [34]. These challenges can be addressed by the scheme in [35]. Even though the authentication protocol in [35] offers efficient data aggregation, it is defenseless against MitM attacks due to lack of mutual authentication procedures. To protect against outsider and insider threats, an authorization and authentication scheme is developed in [36]. Alternatively, a protocol based on identities is introduced in [37] to boost the smart grid security levels. However, the protocol in [37] cannot offer anonymity and untraceability. The authors in [38] demonstrate that this scheme is susceptible to numerous attacks, and hence they introduce a novel lightweight protocol to overcome these security issues. Similarly, the key management protocol in [39] guarantees anonymity of the communicating entities. In addition, it has lower computation costs when compared with the scheme developed in [29]. However, the protocol in [39] is never evaluated against attacks such as privileged insider, known session-specific temporary information (KSSTI), spoofing and de-synchronization.

The Physical Unclonable Function (PUF) presents another valuable technology for providing high security levels at relatively low costs. As such, PUF-based protocols have been presented in [40–42] for the smart grids. The scheme in [41] does not necessitate the maintenance of secret keys for message exchanges with the aggregator. However, security schemes based on PUF technology have stability challenges due to the stochastic nature of PUF outputs. Conversely, an authentication scheme based on temporary credentials is introduced in [43] to secure the smart grid demand response. However, this protocol can support only one smart meter and hence has scalability issues. In addition, attackers can impersonate the Utility Center (UC) since it lacks initial verification on the UC side [44]. This setback can be addressed by the self-sovereign verification protocol in [7] as well as the anonymous authentication protocol in [45]. Although the protocol in [7] protects against masquerading and identity theft, the scheme in [45] is susceptible to DoS attacks [28]. To provide mutual authentication in smart grids, authors in [46] have introduced a pair-wise key generation scheme. However, this approach cannot uphold anonymity and confidentiality [28]. This problem is addressed by the password-based anonymous key agreement protocol in [47], which provides strong mutual authentication, confidentiality, anonymity, perfect forward key secrecy and non-traceability. Unfortunately, this scheme has some design flaws regarding its two points multiplication over the elliptic curve [48]. In addition, it cannot uphold session key security. Further, it is shown to be vulnerable to passive and active attacks which limit its applicability [40]. As such, an improved AKA protocol has been developed in [48]. Although this scheme offers mutual authentication and session key agreement, it is never evaluated against any attack vectors. In addition, it incorporates timestamps during mutual authentication and hence is vulnerable to de-synchronization attacks.

To boost the security in some of the two-factor authentication protocols discussed above, ECC-based AKA techniques have been introduced in [27, 28, 47–52] for industrial smart grids. However, the deployed ECC requires high communication and computation costs [14]. On the other hand, a masked symmetric key-based protocol is developed in [53]. However, attacks such as privileged insider, masquerading and replay are not considered in this scheme. Similarly, a symmetric key and message authentication code-based approach is presented in [54]. Unfortunately, the timestamp deployed in this protocol can potentially lead to clock synchronization problems [55]. This challenge is solved by the scheme in [56], even though this technique cannot verify two entities of the smart grid [57]. Alternatively, the three-factor user validation scheme developed in [51] is vulnerable to stolen mobile device and impersonation attacks [52]. As such, the authors in [52] have introduced an enhanced three-factor AKA protocol to overcome these challenges. Similarly, an AKA scheme for securing demand response is presented in [58]. Unfortunately, since this technique is based on the traditional power grid system, it is inefficient and is unable to offer demand response records integrity. Based on the above discussion, it is clear that most of the current schemes for security and privacy protection in the smart grids have numerous challenges that render them unsuitable for deployment in smart grids. In addition, some of the schemes such as the one in [57] require the involvement of third parties during the establishment of secure communication among the smart grid devices. This inadvertently presents a single point of failure and may result in privileged insider attacks when this third turns out to be malicious. On the other hand, most of the Public-Key-Infrastructure (PKI) based schemes such as the ones in [35, 59, 60], and asymmetric key cryptography based schemes require heavy execution and bandwidth requirements. This is obviously not suitable for smart grid components such as smart gas meters. Although ECC-based techniques such the ones in [61, 62] have reduced computation overheads compared with PKI-based schemes, the smart meters still need to carry out computationally extensive operations. Additionally, the scheme in [61] does not consider privacy protection during the AKA procedures [55]. On its part, the Diffie-Hellman and digital signature-based scheme in [63] incurs high computation and communication costs during signature generation and verification. On the other hand, anonymous authentication scheme is presented in [64] while efficient security protocols are developed in [65, 66]. Conversely, multi-factor authentication schemes are presented in [67, 68] while a blockchain-based protocol is introduced in [69]. However, the protocols in [64–66, 68] have not been evaluated against attacks such as de-synchronization and KSSTI. Similarly, the scheme in [67] has not been evaluated against attacks such as forgery and privileged insider. On the other hand, the blockchain deployed in [69] renders this scheme inefficient [70]. Consequently, the development of novel AKA protocols to address these challenges cannot be overemphasized.

To overcome some of the above challenges, this paper leverages on ECC, Hamming distance and fuzzy extraction to develop a provably secure security scheme. The biometric authentication is facilitated by fuzzy extraction and Hamming distance, while ECC helps in the generation of the random nonces. It is shown that biometric authentication renders our protocol robust against attacks such as privileged insider, guessing and masquerading. On the other hand, the ECC-facilitated random nonces offer untraceability and anonymity. They also help defend against attacks such as de-synchronization, man-in-the-middle (MitM), KSSTI and forgery. These cryptographic primitives are also demonstrated to be lightweight compared to other approaches such as PKI and blockchain.

## 3. System model

This section describes some mathematical preliminaries, threat model as well as the security requirements of the proposed scheme.

### A. Mathematical preliminaries

The proposed security scheme is based on ECC, Hamming distance and fuzzy extraction. Compared with other public-key cryptosystems such as Rivest, Shamir and Adleman (RSA), ECC provides equivalent security level but with shorter key sizes. This is beneficial to resource-limited setting [62] exampled by Internet of Things (IoT) and many smart grid edge devices. In this section, we introduce some mathematical formulations for the basic building blocks of the proposed protocol. This includes hamming distance, fuzzy extraction and elliptic curve cryptography formulations as discussed below.

#### 1) Hamming distance

The Hamming distance plays a critical role during the exchange of information over noisy communication media. During this process, the following 3 definitions hold:

**Definition 1**: Suppose that the sender wants to forward message *Msg = {0*, *1}*^*q*^. The Hamming distance comes handy in fuzzy commitment procedures that facilitate correct transmission of *Msg* to the receiver. Here, the error correction code (*E*_CC_) comprises of code-phrases *CS* ⊆ {0, 1}^ℕ^. At the sender, message *Msg*_i_ ∈ *Msg* is mapped to an element in *CS* before being transmitted.

**Definition 2**: For increased levels of redundancy, *ℕ > q*. A typical *E*_*CC*_ has two functions, for translation and decoding. Let us denote translation function as *t* and decoding function as *d*. As such:

t:Msg→CS

$$d:{\left\{0,1\right\}}^{\mathbb{N}}\to \text{CS}\;\cup \;\left\{\Omega \right\}$$

In this regard, *d* maps ℕ—bit string *s* to the nearest code-phase in *CS* in terms of the Hamming distance (*H*_*D*_). If this mapping is not successful, then *d* outputs *Ω*.

**Definition 3**: Suppose that *d* has a correction threshold of *C*_T_. Then, for some code-phrase *RC* ∈ *CS*, error term *E*_T_ ∈ {0, 1}^ℕ^ with hamming weight ||*E*_T_|| ≤ *C*_T_, the mapping is executed as follows:

$$d(RC⊕E_{T})=RC$$

#### 2) Fuzzy extraction

During biometric authentication process, the fuzzy commitment procedures play a crucial role in validating the input template. In this regard, the following definitions are critical.

**Definition 4**: Suppose that *h*: {0,1}^ℕ^ → {}^ℙ^ is a secure hash function. Taking *W* as an ℕ—bit witness, then the sender can deploy the fuzzy commitment technique *F*: ({0,1}^ℕ^, ({0,1}^ℕ^) → ({0,1}^ℙ^, ({0,1}^ℕ^) to commit a code-phrase *RC* ∈ *CS* using *W* as *F* (*RC*,*W*) = (*ē*, *ū*). Here, *ē = h* (*RC*) and *ū = W*⊕*RC*.

**Definition 5**: Provided that W* is fairly close to *W*, then it can be deployed to decipher commitment *F* (*RC*,*W*) = (*ē*, *ū*). In essence, W* need to be exactly equivalent to *W*. For any successful deciphering, the receiver need to derive RC* = *f* (W*⊕*ū*). Since *ū = W*⊕*RC*, then RC* = *f* (W*⊕*ū*) = *f* (RC⊕(W*⊕*W*)).

**Definition 6**: To validate the sender, the receiver checks whether *ē* ≟ *h* (RC*). Upon successful verification, the deciphering process is treated as being valid. Otherwise, W*is treated as malicious and the sender is flagged as such.

**Definition 7**: In the field of biometric authentication, the input biometric data *Bio*_i_* is not always exactly similar to the biometric template *Bio*_i_. This constitutes noise in this data and hence can be deployed in the fuzzy commitment procedures. In essence, *Bio*_i_ is treated as *W* and therefore *RC* can be deciphered using input biometric W* that is fairly close to *W*.

#### 3) Elliptic curve cryptography

Suppose that *Z*_p_ = {*0*, *1*,*…*, *p − 1*}, where *p > 3*. We also let *δ*_1_, *δ*_2_ ∈ *Z*_p_ be constants such that $4\delta_{1}^{3}+27\delta_{2}^{2}\neq 0$ (mod *p*). Then, the definitions below describe the elliptic curve properties and procedures:

**Definition 8**: A non-singular elliptic curve (*EC*)*y*^*2*^ = *s*^*3*^*+ δ*_*1*_*s+ δ*_*2*_ over the Galois finite field *GF*(*p*) is a set *E*_p_ (*δ*_1_, *δ*_2_) of the solutions (*s*, *y*) ∈ *Z*_p_ × *Z*_p_ to the congruence *y*^*2*^ *≡ s*^*3*^*+ δ*_*1*_*s+ δ*_*2*_ (mod *p*).

**Definition 9**: Suppose that *P* = (*s*_*P*_, *y*_*P*_), *Q* = (*s*_*Q*_, *y*_*Q*_) ∈ *E*_p_ (*δ*_*1*_, *δ*_*2*_) and Ø is the zero point or point at infinity. Under this condition, *s*_*Q*_ = *s*_*P*_ and *y*_*Q*_ = -*y*_*p*_ when *P + Q = Ø*. In addition, *P + Ø = Ø + P = P*, for all *P* ∈ *E*_p_ (*δ*_*1*_, *δ*_*2*_).

**Definition 10**: Taking # *ǹ* as the number of points on *E*_p_ (*δ*_*1*_, *δ*_*2*_), then in accordance with Hasse’s theory, the inequality below holds:

P+1-2√p≤#ǹ≤p+1+2√p

This means that there are *p* points on *E*_p_ (*δ*_*1*_, *δ*_*2*_) over *Z*_p_. Additionally, *E*_p_ (*δ*_*1*_, *δ*_*2*_) constitute commutative group in accordance with addition modulo *p* operation.

**Definition 11**: In accordance with the *EC* point addition, we take *P* and *Q* as two points on the *EC E*_p_ (*δ*_*1*_, *δ*_*2*_). Then *Ǻ = (s*_*Ǻ*_, *y*_*Ǻ*_*) = P + Q* is derived as described below:

$$s_{Ǻ}=\left(\sigma^{2}-s_{P}-\;s_{Q}\right)(\text{mod}\;p)$$

$$y_{Ǻ}=\left(\sigma \left(s_{P}-s_{Ǻ}\right)-y_{P}\right)(\text{mod}\;p),$$

where

σ=yQ-yPsQ-sPmodp,forP≠-Q3sP2+δ12yPmodp,forP=Q

**Definition 12**: Based on the elliptic curve point multiplication (scalar multiplication), we let *P* ∈ *E*_p_ (*δ*_*1*_, *δ*_*2*_). Then, *6P* implies repeated additions such that 6*P* = *P* + *P* + *P* + *P* + *P* + *P*.

### B. Threat model

The Dolev-Yao (DY) and Canetti- Krawczyk (CK) threat models are frequently utilized in the semantic evaluation of authentication schemes. Therefore, this paper adopts these two threat models, in which it is assumed that adversary *Å*:

1. Can forge, delete, reuse, change, insert, block and eavesdrop the messages exchanged across the insecure communication channels.
2. Has the potential and capability of physically accessing the smart grid components such as the smart meter and extract secret security tokens stored in its memory. This is facilitated using techniques such as side-channeling.
3. Upon obtaining the smart meter security parameters, *Å* can launch attacks such as spoofing, forgery, guessing, de-synchronization, KSSTI, replay, masquerading, man-in-the-middle and privileged insider.
4. Can capture session keys as well as their intermediary states.

### C. Security requirements

To provide enhanced message protection in the smart grid environment, the following goals need to be fulfilled:

1. *Anonymity*: This property ensures that user and smart meter real identities cannot be discerned from any messages captured over the transmission channel.
2. *Untraceability*: An attacker who successfully intercepts the message exchange process between the smart meter and the smart grid server should not be in a position to associate any communication sessions to a specific user or smart meter.
3. *Mutual Authentication*: All the communicating entities should verify each other’s identity before they can commence message exchanges.
4. *Session Key Negotiation*: After successfully validation procedures, the smart grid entities must agree on some shared key that they will utilize to protect the exchanged messages.
5. *Attacks Resilience*: It should be cumbersome for the adversary to launch common smart grid attacks such privileged insider, spoofing, physical capture, smart card loss, forgery, replay, masquerading, de-synchronization, guessing, KSSTI, and man-in-the-middle attacks.

## 4. The proposed protocol

**Fig 1:**
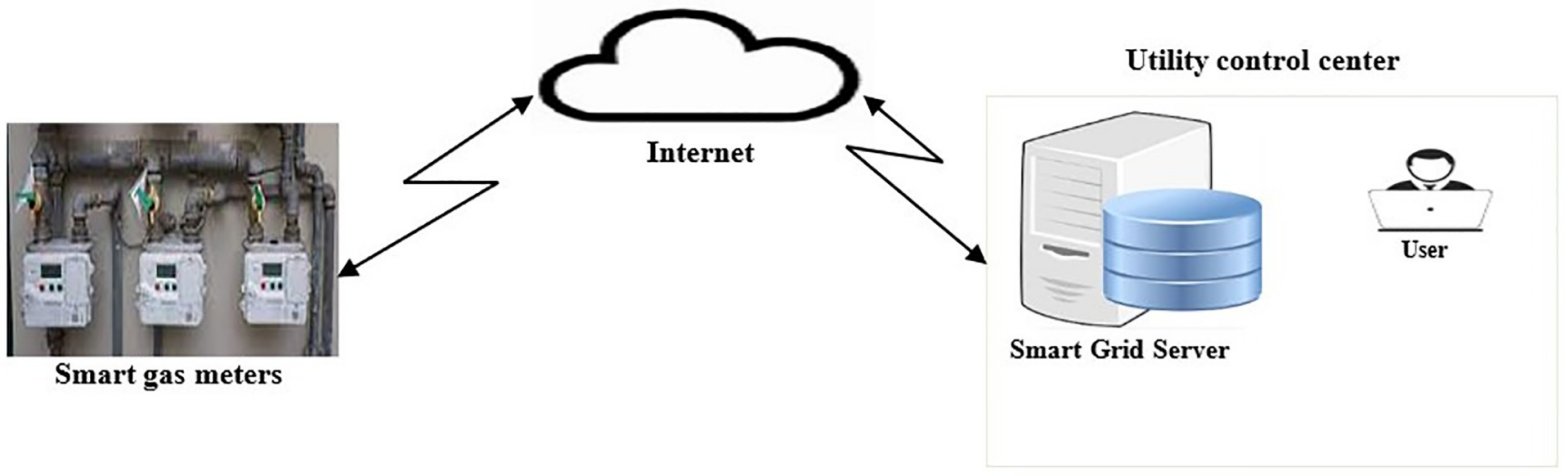
Network architecture.

The proposed protocol involves three entities which include the Smart Meter (*SM*_i_), User (*U*_i_) and Smart Grid Server (*SGS*_i_) as shown in Fig 1. Here, the *SM*_i_ collects energy consumption information from the consumers and forwards the same to the *SGS*_i_ located at the utility control center. As illustrated in Fig 1, the communication channel between *SM*_i_ and *SGS*_i_ is the public internet.

Depending on the received consumption reports from the *SM*_i_, decisions are made by the *SGS*_i_ regarding energy adjustments. In this context, the *U*_i_ refers to the system administrator located at the utility control center. Table 1 details the symbols used in this paper.

**Table 1:** Deployed symbols.

Symbol	Description
*F*_p_	Elliptic curve *E* finite field
*G*	Additive group over *F*_p_
*SM*_i_	Smart meter *i*
*SMID*_i_	Unique identity of *SM*_i_
*SGS*_i_	Smart grid server *i*
*U*_i_	User *i*
*R*_i_	Random nonce *i*
*P*_K_	*R*_i_’s corresponding public key
*SGS*_MK_	Smart grid server master key
*SK*_S-SM_	Secret key shared between *SGS*_i_ and *SM*_i_
*UID*_i_	User identity
*PW*_i_	User password
*Bio*_i_	User biometric data
*U*_SC_	User’s smart card
*ɸ*_M_	Session key computed at the *SM*_i_
*ɸ*_S_	Session key calculated at the *SGS*_i_
*ɸ*_U_	Session key derived by *U*_i_
||	Concatenation operation
⊕	XOR operation

From the execution perspective, this scheme comprises of 7 phases: system initialization, smart meter registration, user registration, login, authentication, session negotiation, and finally password change. The sub-sections below discuss the details of each of these phases.

### A. System initialization

In this scheme, the smart grid server *SGS*_i_ is a trusted entity that bridges the communication between the smart meter *SM*_i_ and user *U*_i_. During the initialization phases, the security tokens applicable during the subsequent registration, login, authentication and key establishment phases are derived.

**Step 1**: The *SGS*_i_ selects elliptic curve additive group *G* over some finite field *F*_p_, where *P* is the generator, whose order is a large prime number *ℕ*.

**Step 2**: The *SGS*_i_ generates random nonce $R_{1}\in Z_{N}^{*}$ and sets it as its clandestine key. Next, the *SGS*_i_ derives its corresponding public key *P*_K_ = *PR*_1_. Next, the *SGS*_i_ chooses *SGS*_MK_ as its master key. The keys *R*_1_ and *SGS*_MK_ will be used later during the registration, login and authentication phases to derive ephemeral parameters.

**Fig 2:**
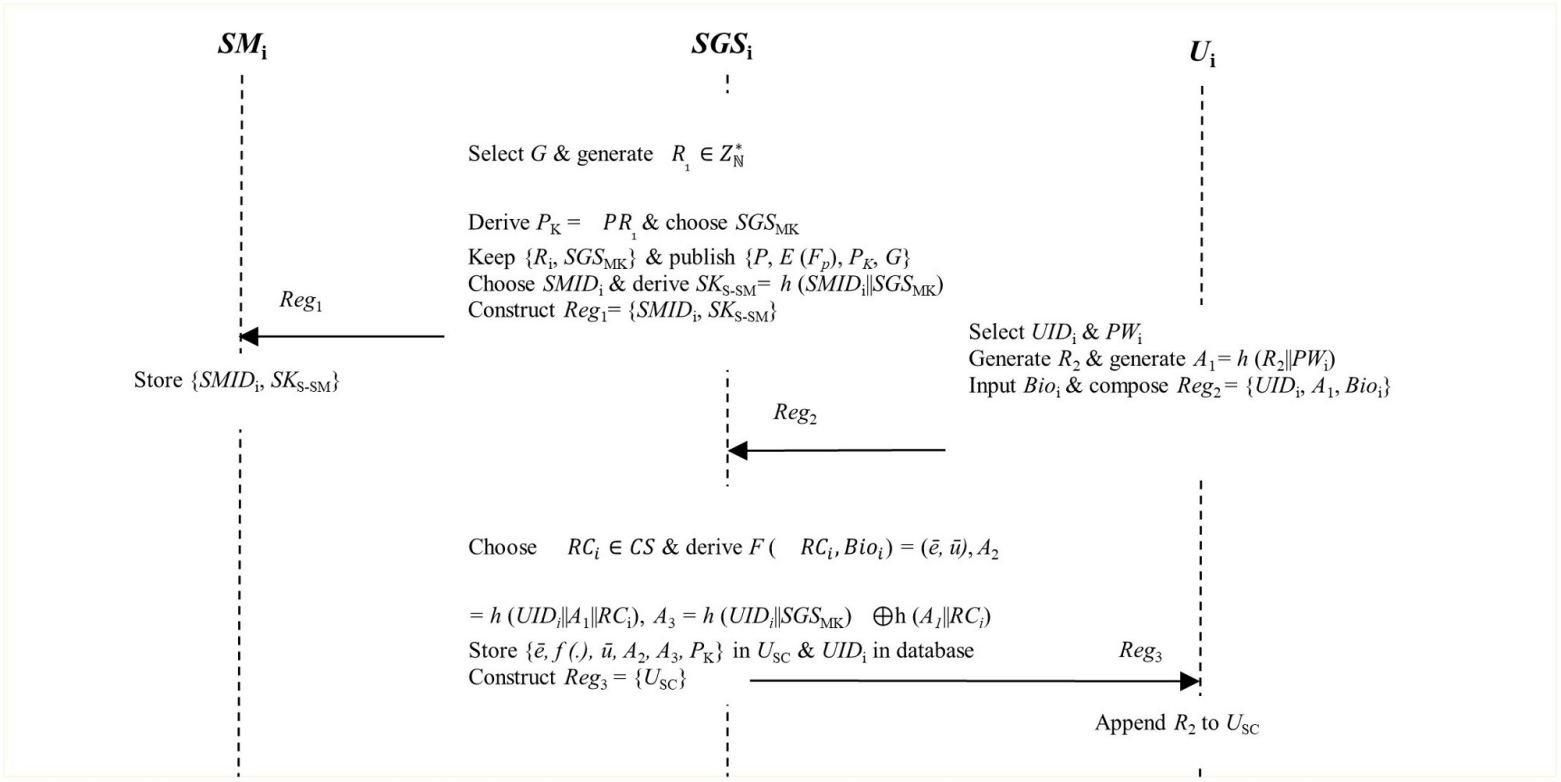
System initialization and registration phases.

**Step 3**: Security parameters *R*_1_ and *SGS*_MK_ are secretly kept by the *SGS*_i_ in its database. Next, it publishes parameter set {*P*, *E* (*F*_*p*_), *P*_*K*_, *G*}. All these security parameters are deployed by the network entities to compute intermediary tokens during the registration, login, authentication and key negotiation phases. Fig 2 presents the diagrammatical depiction of these steps.

### B. Smart meter registration

Before the deployment of the smart meters, they must generate and be assigned security parameters that they store in their memories. These parameters are then used during login phase and also to authenticate these smart meters to the smart grid network. To realize this, the two steps described below are executed.

**Step 1**: The smart grid server *SGS*_i_ chooses value *SMID*_i_ as the identity of smart meter *SM*_i_. Next, it derives *SK*_S-SM_ = *h* (*SMID*_i_||*SGS*_MK_) as the secret key between *SGS*_i_ and *SM*_i_ as shown in Fig 2.

**Step 2**: The *SGS*_i_ composes registration message *Reg*_1_ = {*SMID*_i_, *SK*_S-SM_} which is forwarded to the *SM*_i_ for private buffering in its memory. Finally, smart meter *SM*_i_ is deployed in particular premises.

### C. User registration

In smart grids, the users may be interested in accessing the smart grid data deployed in some given premises. It is therefore important that all users be registered at the smart grid server *SGS*_i_ as shown in Fig 2. This registration is important as the users are assigned parameters that they will deploy during the subsequent login, authentication and session key establishment phases. This phase is executed using the five steps described below.

**Step 1**: The user *U*_i_ chooses some unique user identity *UID*_i_ and strong password *PW*_i_. Next, the user *U*_i_ generates random nonce *R*_2_ that is used to derive parameter *A*_1_ = *h* (*R*_2_||*PW*_i_).

**Step 2**: The user imprints biometric *Bio*_i_ on the reader device. Next, the user registration request *Reg*_2_ = {*UID*_i_, *A*_1_, *Bio*_i_} is composed and transmitted over to the *SGS*_i_ across secure communication channels.

**Step 3**: After getting registration request *Reg*_2_ from *U*_i_, the *SGS*_i_ selects some random code-phrase *RC*_*i*_ ∈ *CS* for this particular user. Next, it derives *F* (*RC*_*i*_, *Bio*_*i*_) = *(ē*, *ū)*, where *ē = h* (*RC*_*i*_) and *ū = RC*_*i*_ ⊕ *Bio*_*i*_.

**Step 4**: The *SGS*_i_ derives parameter *A*_2_ = *h* (*UID*_*i*_||*A*_1_||*RC*_i_) and *A*_3_ = *h* (*UID*_*i*_||*SGS*_MK_)⊕h (*A*_*1*_||*RC*_*i*_). Afterwards, the *SGS*_i_ stores parameter set {*ē*, *f* (.), *ū*, *A*_2_, *A*_3_, *P*_K_} in *U*_i_’s smart card *U*_SC_. Next, it composes registration response message *Reg*_3_ = {*U*_SC_} that is sent to *U*_i_ over secure channels. Finally, the *SGS*_i_ buffers *UID*_i_ in its database.

**Step 5**: On getting this smart card, the user appends *R*_2_ into it. As such, the smart card now holds parameter set {*ē*, *f* (.), *ū*, *A*_*2*_, *A*_*3*_, *P*_*K*_, *R*_2_}. Algorithm 1 below summarizes the system initiation, user registration and smart meter registration processes.

**Algorithm 1. System Initiation, User and Smart Meter Registration**

***Begin***

# ***System Initialization***

Choose *G* over *F*_p_ and generate random nonce *R*_1_ as private key

Derive corresponding public key as *P*_K_ and master key as *SGS*_MK_

Secretly keep *R*_1_ and SGS_MK_ at *SGS*_i_ and publish parameter set {*P*, *E* (*F*_p_), *P*_K_, *G*}

#****Smart Meter Registration***

Choose *SMID*_i_ as smart meter *SM*_i_’s identity

Set *SK*_S-SM_ as secret key between smart grid server *SGS*_i_ and smart meter *SM*_i_

Privately store parameter set {*SMID*_i_, *SK*_S-SM_}in smart meter *SM*_i_’s memory

Deploy *SM*_i_ in its application domain

#***User Registration***

Select user identity and password as *UID*_i_ and *PW*_i_ respectively

Generate random nonce *R*_2_ and derive *A*_1_

Imprint user biometric *Bio*_i_ into the reader, compose registration message *Reg*_1_ then forward it to *SGS*_i_

Select random code-phrase *RC*_i_ then derive values *F* (*RC*_i_, Bio_i_), *A*_2_ and *A*_3_

Store values {*ē*, *f* (.), *ū*, *A*_2_, *A*_3_, *P*_K_} in smart card *U*_SC_

Construct registration message *Reg*_2_ then forward it to user *U*_i_

Buffer user identity *UID*_i_ in smart grid server *SGS*_i_’s database

Append random nonce *R*_2_ to smart card *U*_SC_

***End***

### D. Login, mutual authentication and session negotiation phase

The aim of this phase is to ensure that all users and smart meters accessing the smart grid server are legitimate entities and hence protect the network against attacks. In addition, the session key is derived which will be used to encipher the data exchanged among the users, smart meters and smart grid servers. To accomplish this, user biometric data *Bio*_i_*, password *PW*_i_, user identity *UID*_i_ and smart card *U*_SC_ are deployed. Here, the *U*_SC_ buffers the security tokens that the user utilizes to derive ephemeral security parameters. This is an 8 step process as described below. Algorithm 2 gives the summary of the login, mutual authentication and session negotiation phase.

**Step 1**: The user the inserts *U*_SC_ into its reader device before imprinting biometric *Bio*_i_*. Thereafter, the smart card computes $R{C_{\text{i}}}^{*}=f(ū⊕Bio_{\text{i}}^{*})$. Since *ū = RC*_*i*_⊕*Bio*_*i*_), then *RC*_i_* can also be expressed as $R{C_{\text{i}}}^{*}=f(RC_{\text{i}}⊕(Bio_{i}⊕Bio_{\text{i}}^{*})$.

**Step 2**: The smart card confirms whether *h* (*RC*_i_*) ≟*ē = h* (*RC*_i_). Essentially, the session is aborted whenever this verification flops. This means that the user has to initiate another login attempt. Otherwise, this particular user has successfully passed the biometric authentication.

**Step 3**: The user inputs identity *UID*_i_ and password *PW*_i_ after which parameter *A*_2_* = *h* (*UID*_*i*_|| *h (R*_2_||*PW*_i_||*RC*_i_*) is derived. This is followed by the checking of whether *A*_2_* ≟ *A*_*2*_ such that the session is aborted when these two parameters are not equivalent. Consequently, the user must re-enters the correct values for *UID*_i_ and *PW*_i_. Otherwise, the user’s *UID*_i_ and *PW*_i_ have been successfully verified by the smart card.

**Fig 3:**
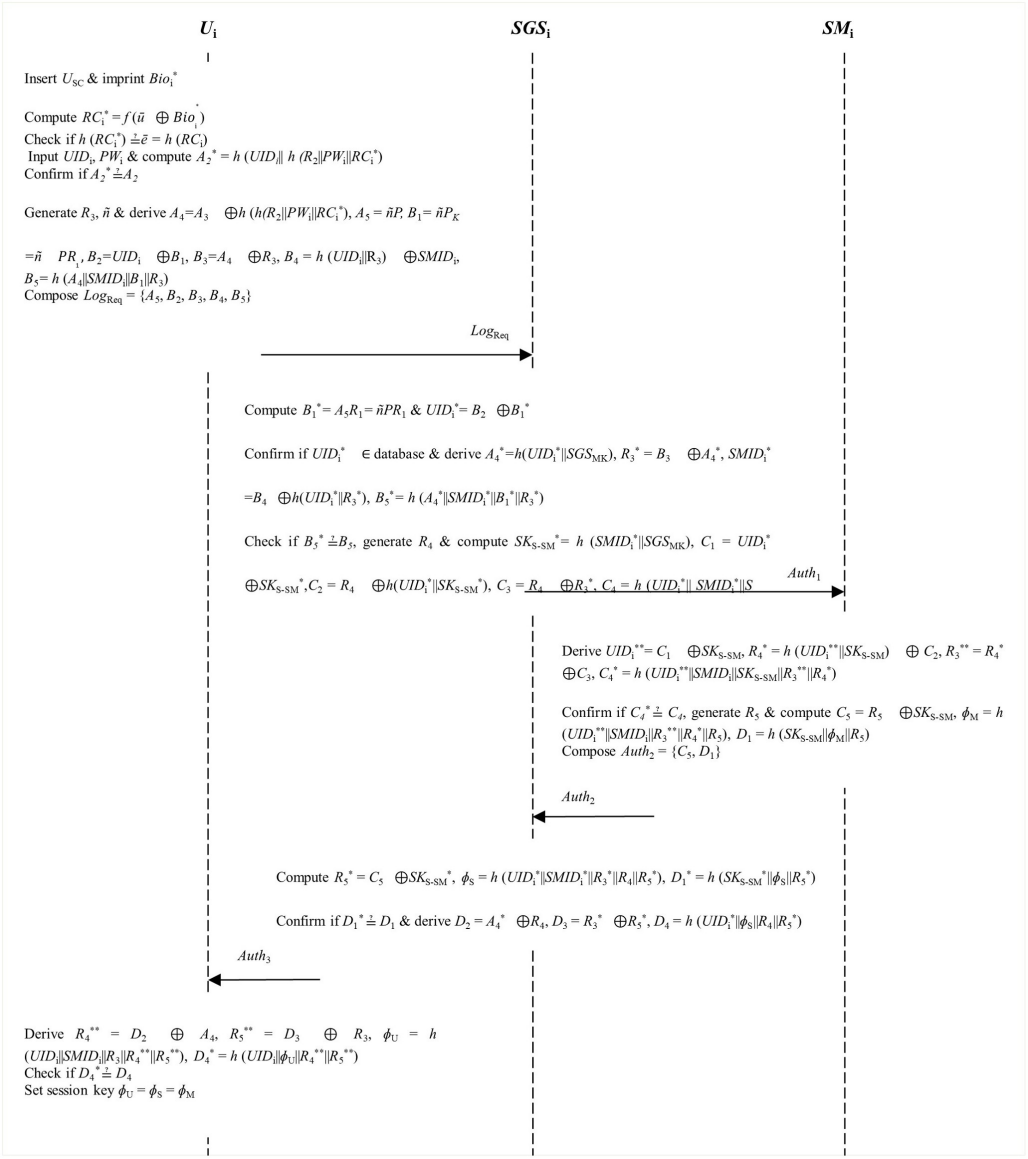
Login, authentication and session negotiation phase.

**Step 4**: The *U*_SC_ chooses some arbitrary nonce *R*_3_ and parameter $ñ\in Z_{\mathbb{N}}^{*}$. Next, it computes security parameters *A*_4_ = *A*_3_⊕*h* (*h*(*R*_2_||*PW*_i_||*RC*_i_*), *A*_5_ = *ñP*, *B*_1_ = *ñP*_*K*_ = *ñPR*_1_, *B*_2_ = *UID*_i_⊕*B*_1_, *B*_3_ = *A*_4_⊕*R*_3_, *B*_4_ = *h* (*UID*_i_||R_3_)⊕*SMID*_i_ and *B*_5_ = *h* (*A*_4_||*SMID*_i_||*B*_1_||*R*_3_). Finally, it constructs login request message *Log*_Req_ = {*A*_5_, *B*_2_, *B*_3_, *B*_4_, *B*_5_} that is sent to the *SGS*_i_ as illustrated in Fig 3.

**Step 5**: On receiving login request *Log*_Req_, the *SGS*_i_ computes parameters *B*_1_* = *A*_5_*R*_1_ = *ñPR*_1_ and *UID*_i_* = *B*_2_⊕*B*_1_*. Next, it confirms whether the derived identity *UID*_i_* is in its database such that the login request is rejected if it is not. When this happens, the *SGS*_i_ must request the user to resend valid message *Log*_Req_. Otherwise, the *SGS*_i_ computes *A*_4_* = *h*(*UID*_i_*||*SGS*_MK_), *R*_3_* = *B*_3_⊕*A*_4_*, *SMID*_i_* = *B*_4_⊕*h*(*UID*_i_*||*R*_3_*) and *B*_5_* = *h* (*A*_4_*||*SMID*_i_*||*B*_1_*||*R*_3_*). It then checks whether *B*_5_* ≟ *B*_*5*_ such that the session is aborted if these parameters are not identical. Once again, the *SGS*_i_ prompts the user to re-forward valid message *Log*_Req_. Otherwise, the *SGS*_i_ generates some arbitrary nonce *R*_4_ that it deploys to re-compute secret key *SK*_S-SM_* = *h* (*SMID*_i_*||*SGS*_MK_), *C*_1_ = *UID*_i_*⊕*SK*_S-SM_*, *C*_2_ = *R*_4_⊕*h*(*UID*_i_*||*SK*_S-SM_*), *C*_3_ = *R*_4_⊕*R*_3_* and *C*_4_ = *h* (*UID*_i_*|| *SMID*_i_*||*SK*_S-SM_*||*R*_3_*||*R*_4_). Finally, it composes authentication message *Auth*_1_ = {*C*_1_, *C*_2_, *C*_3_, *C*_4_} which it forwards to the *SM*_i_.

**Step 6**: Upon getting hold of message, the *SM*_i_ derives *UID*_i_** = *C*_1_⊕*SK*_S-SM_, *R*_4_* = *h* (*UID*_i_**||*SK*_S-SM_)⊕ *C*_2_, *R*_3_** = *R*_4_*⊕*C*_3_ and *C*_4_* = *h* (*UID*_i_**||*SMID*_i_||*SK*_S-SM_||*R*_3_**||*R*_4_*). It then checks whether *C*_4_* ≟ *C*_*4*_ such that the session is aborted on condition that this validation flops. Here, *SM*_i_ requests the *SGS*_i_ to resend valid login message *Auth*_1_. Otherwise, the *SM*_i_ generates arbitrary nonce *R*_5_ that it utilizes to compute *C*_5_ = *R*_5_⊕*SK*_S-SM_, *ɸ*_M_ = *h* (*UID*_i_**||*SMID*_i_||*R*_3_**||*R*_4_*||*R*_5_) and *D*_1_ = *h* (*SK*_S-SM_||*ɸ*_M_||*R*_5_). At the end, the *SM*_i_ composes authentication message *Auth*_2_ = {*C*_5_, *D*_1_} that is sent to the *SGS*_i_.

**Step 7**: Once it has obtained message *Auth*_2_, the *SGS*_i_ derives *R*_5_* = *C*_5_⊕*SK*_S-SM_*, *ɸ*_S_ = *h* (*UID*_i_*||*SMID*_i_*||*R*_3_*||*R*_4_||*R*_5_*) and *D*_1_* = *h* (*SK*_S-SM_*||*ɸ*_S_||*R*_5_*). Next, it confirms whether *D*_1_* ≟ *D*_*1*_ such that the session is rejected when this validation flops. When this happens, *SGS*_i_ must prompt the *SM*_i_ to re-forward valid authentication message *Auth*_2_. Otherwise, the *SGS*_i_ derives *D*_2_ = *A*_4_*⊕*R*_4_, *D*_3_ = *R*_3_*⊕*R*_5_* and *D*_4_ = *h* (*UID*_i_*||*ɸ*_S_||*R*_4_||*R*_5_*). Finally, the *SGS*_i_ constructs authentication message *Auth*_3_ = {*D*_2_, *D*_3_, *D*_4_} that is forwarded to user *U*_i_.

**Step 8**: Upon obtaining message *Auth*_3_, user *U*_i_ computes *R*_4_** = *D*_2_ ⊕ *A*_4_, *R*_5_** = *D*_3_ ⊕ *R*_3_, *ɸ*_U_ = *h* (*UID*_i_||*SMID*_i_||*R*_3_||*R*_4_**||*R*_5_**) and *D*_4_* = *h* (*UID*_i_||*ɸ*_U_||*R*_4_**||*R*_5_**). Thereafter, it validates whether *D*_4_* ≟ *D*_*4*_ such that the session is terminated whenever this verification fails. Once again, the user has to prompt the *SGS*_i_ for the correct authentication message *Auth*_3_.

**Algorithm 2. Login, Mutual Authentication and Session Negotiation**

***Begin***

#***Login***#

Insert *U*_SC_ to the card reader and imprint *Bio*_i_*

Compute *RC*_i_*

**IF** *h* (*RC*_i_*) ! = ē ! = *h* (*RC*_i_) **THEN**:

 Terminate session

**ELSE**: Biometric authentication passed

**ENDIF**

Input *UID*_i_, *PW*_*i*_ and derive *A*_2_*

**IF** *A*_2_* ! = *A*_2_ **THEN**:

 Abort session

**ELSE**: *UID*_i_, *PW*_i_ have passed verification

Select *R*_3_, *ñ* and derive *A*_4_, *A*_5_, *B*_1_, *B*_2_, *B*_3_, *B*_4_ & *B*_5_

Construct *Log*_Req_ and forward it to *SGS*_i_

Derive *B*_1_*and *UID*_i_*

**IF** *UID*_i_* is not in database **THEN**:

  Deny login request

#***Mutual authentication and session negotiation***#

**ELSE**: compute *A*_4_*, *R*_3_*, *SMID*_i_* and *B*_5_*

**IF** *B*_5_* ! = *B*_5_**THEN**:

   Abort session

**ELSE**: Generate *R*_4_ and derive *SK*_S-SM_*, *C*_1_, *C*_2_, *C*_3_ and *C*_4_

 Compose *Auth*_1_ and forward it to *SM*_i_

 Derive *UID*_i_**, *R*_4_*, *R*_3_** and *C*_4_*

 **IF** *C*_4_* ! = *C*_4_**THEN**:

  Terminate session

 **ELSE**: Generate *R*_5_ and compute *C*_5_, *ɸ*_M_ & *D*_1_

  Compose *Auth*_2_ and forward it to *SGS*_i_

  Derive *R*_5_*, *ɸ*_S_ & *D*_1_*

  **IF** *D*_1_* ! = *D*_1_**THEN**:

   End session

  **ELSE**: Compute *D*_2_, *D*_3_ & *D*_4_

   Construct *Auth*_3_ and forward it to *U*_i_

   Derive *R*_4_**, *R*_5_**, *ɸ*_U_ and *D*_4_*

   **IF** *D*_4_* ! = *D*_4_ **THEN**:

    Terminate session

   **ELSE**: Set session key as *ɸ*_U_ = *ɸ*_S_ = *ɸ*_M_

   ENDIF; ENDIF; ENDIF;

 ENDIF; ENDIF; ENDIF;

***End***

Otherwise, the user, smart meter and the smart grid server have successfully authenticated each other. In addition, these three entities share session key *ɸ*_U_ = *ɸ*_S_ = *ɸ*_M_. As such, the user can now access the smart meter data through the smart grid server.

In Algorithm 2, the expected outcomes or state changes in each step of Algorithm 2 are the various transient security parameters derived during the login, mutual authentication and session negotiation procedures.

### E. Password change phase

This stage is triggered whenever the user password is compromised. It can also be triggered if the security policy advocates for periodic password change. The following 4 steps are executed during this phase. Algorithm 3 summarizes the password change phase.

**Step 1**: The user *U*_i_ inserts *U*_SC_ into its reader and imprints biometric data *Bio*_i_* on the special device. Afterwards, the smart card derives parameter *RC*_i_*and confirms whether *h* (*RC*_i_*)≟ *ē = h*(*RC*_i_). Here, the session is aborted if this verification flops. When this occurs, the user is prompted to re-enter correct values for the biometric data *Bio*_i_*.

**Step 2**: Provided that the validation in *Step 1* is successful, the user has successfully passed the biometric confirmation process. Next, the *U*_i_ inputs *UID*_*i*_ and *PW*_i_ upon which parameter *A*_2_* is derived.

**Step 3**: The *U*_SC_ validates the derived parameter *A*_2_* against *A*_2_ such that the password change request is turned down if these two parameters are disparate. At this juncture, the user is prompted to re-enter correct values for *UID*_*i*_ and *PW*_i_. However if they are equivalent, the *U*_SC_ permits *U*_i_ to input new password *PW*_i_^New^.

**Step 4**: The *U*_SC_ derives security parameters *A*_2_^New^ = *h* (*UID*_*i*_||*h(R*_2_||*PW*_i_^New^)||*RC*_i_*) and *A*_3_^New^ = *A*_3_ ⊕ *h*(*h*(*R*_2_||*PW*_i_)||*RC*_i_*) ⊕ *h*(*h*(*R*_2_||*PW*_i_^New^)||*RC*_i_*). Finally, the smart card substitutes security parameter set {*A*_2_, *A*_3_} with their refreshed counterparts {*A*_2_^New^, *A*_3_^New^}.

**Algorithm 3. Password Change**

***Begin***

 Insert *U*_SC_ into the card reader and imprint *Bio*_i_*

Derive *RC*_i_*

**IF** *h* (*RC*_i_*)! = *ē*! = *h*(*RC*_i_) **THEN**:

 Abort session

**ELSE**: Biometric authentication passed

 Input *UID*_i_ and *PW*_i_

 Derive *A*_2_*

 **IF** *A*_2_* ! = *A*_2_**THEN**:

  Reject password change request

 **ELSE**: Permit *U*_i_ to input *PW*_i_^New^

  Derive *A*_2_^New^ and *A*_3_^New^

  Substitute {*A*_2_, *A*_3_} with {*A*_2_^New^, *A*_3_^New^}

**ENDIF**

**ENDIF**

***End***

Algorithm 3 is basically a summary of the *Password Change Phase* and hence all the validations and changes occurring are captured in step 1 to step 4 of this phase.

## 5. Security analysis

In this section, the most outstanding security characteristics offered by our scheme are both formally and semantically analyzed as described below.

### A. Formal security analysis

To show that strong mutual authentication and common session keys negotiation are carried out among the *U*_i_, *SGS*_i_ and *SM*_i_, the Burrows-Abadi-Needham logic (BAN logic) is deployed. Table 2 presents the BAN logic notations deployed during this proof.

**Table 2:** BAN logic notations.

Notation	Descriptions
*A*| ≡ *B*	*A* trusts *B*
$A\overset{K}{↔}D$	*A* and *D* deploy shared key to communicate
*A* ⨞ *B*	*A* sees *B*, meaning that A has received *B*
{*B,C*}_*K*_	Statements *B* and *C* are hashed with *K*
*A*| ~*B*	*A* once said *B*, meaning *A* had sent message *B*
〈*B*〉_*K*_	*B* is enciphered with *K*
*A* ⇒ *B*	*A* has jurisdiction over *B*
(*B*,*C*)	*B* or *C* is part of message *(B*, *C)*
# (*B*)	*B* is fresh

During the BAN logic analysis, the BAN logic rules in Table 3 are deployed.

**Table 3:** BAN logic rules.

Rule	Description
$\frac{A|\equiv (B),A|\equiv C)}{A|\equiv (B,C)}$	Believe rule (*BR*)
$\frac{A|\equiv A\overset{K}{↔}D,A⨞{\left\{B\right\}}_{K}}{A|\equiv D|\sim B}$	Message-meaning rule (*MMR*)
$\frac{A|\equiv \#(B),A|\equiv D|\equiv B)}{A|\equiv A\overset{K}{↔}D}$	Session key rule (*SKR*)
$\frac{A|\equiv \#\left(B\right),A|\equiv D|\sim B}{A|\equiv D|\equiv B}$	Nonce verification rule (*NVR*)
$\frac{A|\equiv \#(B)}{A|\equiv \#(B,C)}$	Fresh-promotion rule (*FPR*)
$\frac{A|\equiv D\Rightarrow B,A|\equiv D|\equiv B}{A|\equiv B}$	Jurisdiction rule (*JR*)
$\frac{A⨞(B,C)}{A⨞B}$	Seeing Rule (SR)

For strong privacy and security enhancement in smart grids, the eight goals in Table 4 must be satisfied.

**Table 4:** Security goals.

Goal	Description
G-1	$SM_{\text{i}}|\equiv SM_{\text{i}}\overset{{ɸ}_{\text{M}}}{↔}U_{\text{i}}$
G-2	$SM_{\text{i}}|\equiv U_{\text{i}}|\equiv SM_{\text{i}}\overset{{ɸ}_{\text{M}}}{↔}U_{\text{i}}$
G-3	$U_{\text{i}}|\equiv SM_{\text{i}}\overset{{ɸ}_{\text{U}}}{↔}U_{\text{i}}$
G-4	$U_{\text{i}}|\equiv SM_{\text{i}}|\equiv SM_{\text{i}}\overset{{ɸ}_{\text{U}}}{↔}U_{\text{i}}$
G-5	$SGS_{\text{i}}|\equiv SGS_{\text{i}}\overset{{ɸ}_{\text{S}}}{↔}U_{\text{i}}$
G-6	$SGS_{\text{i}}|\equiv U_{\text{i}}|\equiv SGS_{\text{i}}\overset{{ɸ}_{\text{S}}}{↔}U_{\text{i}}$
G-7	$SGS_{\text{i}}|\equiv SGS_{\text{i}}\overset{{ɸ}_{\text{S}}}{↔}SM_{\text{i}}$
G-8	$SGS_{\text{i}}|\equiv SM_{\text{i}}|\equiv SGS_{\text{i}}\overset{{ɸ}_{\text{S}}}{↔}SM_{\text{i}}$

To proof the security goals in Table 4 above, the following initial state assumptions (*ISA*) are made.


$$ISA_{1}:U_{\text{i}}|\equiv \#R_{3}$$

$$ISA_{2}:SGS_{\text{i}}|\equiv \#R_{4}$$

$$ISA_{3}:SM_{\text{i}}|\equiv \#R_{5}$$

$$ISA_{4}:U_{\text{i}}|\equiv U_{\text{i}}\overset{ñPR_{1}}{↔}SGS_{\text{i}}$$

$$ISA_{5}:U_{\text{i}}|\equiv U_{\text{i}}\overset{{ɸ}_{\text{M}}}{↔}SM_{\text{i}}$$

$$ISA_{6}:SGS_{\text{i}}|\equiv SGS_{\text{i}}\overset{ñPR_{\text{1}}}{↔}U_{\text{i}}$$

$$ISA_{7}:SGS_{\text{i}}|\equiv SGS_{\text{i}}\overset{SK_{\text{S}-\text{SM}}}{↔}SM_{\text{i}}$$

$$ISA_{8}:SM_{\text{i}}|\equiv SM_{\text{i}}\overset{{ɸ}_{\text{M}}}{↔}U_{\text{i}}$$

$$ISA_{9}:SM_{\text{i}}|\equiv SM_{\text{i}}\overset{SK_{\text{S}-\text{SM}}}{↔}SGS_{\text{i}}$$

$$ISA_{10}:U_{\text{i}}|\equiv SM_{\text{i}}\Rightarrow R_{5},{ɸ}_{\text{M}}$$

$$ISA_{11}:U_{\text{i}}|\equiv SGS_{\text{i}}\Rightarrow R_{4},{ɸ}_{\text{S}}$$

$$ISA_{12}:SGS_{\text{i}}|\equiv U_{\text{i}}\Rightarrow R_{3},{ɸ}_{\text{U}},ñPR_{\text{1}}$$

$$ISA_{13}:SGS_{\text{i}}|\equiv SM_{\text{i}}\Rightarrow R_{5}⊕SK_{\text{S-SM}}$$

$$ISA_{14}:SM_{\text{i}}|\equiv SGS_{\text{i}}\Rightarrow R_{4}⊕h(UID_{\text{i}}∥SK_{\text{S-SM}})$$

$$ISA_{15}:SM_{\text{i}}|\equiv U_{\text{i}}\Rightarrow R_{3},{ɸ}_{\text{U}}$$

To effectively execute the BAN logic proofs, the messages transmitted during the login, authentication and key negotiation phases are converted into idealized format as follows.

***Log***_**Req**_: *U*_i_ *→ SGS*_i_: {*A*_5_, *B*_2_, *B*_3_, *B*_4_, *B*_5_}

$$\begin{array}{c} \{ñP,{\langle UID_{\text{i}}\rangle }_{ñP_{K}},{\langle R_{3}\rangle }_{h(UID_{\text{i}}\|{\text{SGS}}_{\text{MK}})},{\langle SMID_{\text{j}}\rangle }_{h(UID_{\text{i}}|{|\text{R}}_{3}\text{)}},\\ {\left(SMID_{\text{i}}\|{\text{R}}_{3}\right)}_{ñP_{K}},h(UID_{\text{i}}\|SGS_{\text{MK}})\} \end{array}$$

***Auth***_**1**_: *SGS*_i_ → *SM*_i_: {*C*_1_, *C*_2_, *C*_3_, *C*_4_}

$$\begin{array}{c} \{{\langle {\text{UID}}_{\text{i}}^{*}\rangle }_{SK_{S-SM}},{\langle R_{4}\rangle }_{h({\text{UID}}_{\text{i}}^{*}\|{\text{SG}}_{\text{S}-\text{SM}}^{*})},\\ {\langle R_{3}\rangle }_{R_{4}},{\left(UID_{\text{i}}\|SMID_{\text{i}}\right)}_{\left(R_{3},R_{4},{\text{SG}}_{\text{S}-\text{SM}}^{*}\right)}\} \end{array}$$

***Auth***_**2**_: *SM*_i_ → *SGS*_i_: {*C*_5_, *D*_1_}

$$\left\{{\langle R_{5}\rangle }_{SK_{S-SM}},{\left(R_{5}\right)}_{\left({ɸ}_{M,}{\text{SG}}_{\text{S}-\text{SM}}^{*}\right)}\right\}$$

***Auth***_**3**_: *SGS*_i_ → *U*_i_:{*D*_2_, *D*_3_, *D*_4_}

$$\left\{{\langle R_{4}\rangle }_{h(UID_{\text{i}}\|{\text{SGS}}_{\text{MK}})},{\langle R_{5}^{*}\rangle }_{R_{3}^{*}},{\left(UID_{\text{i}}^{*}\right)}_{\left(R_{4},\;R_{5}^{*},{ɸ}_{S}\right)}\right\}$$

Thereafter, using the above initial state assumptions, BAN logic rules and idealized messages, the above formulated security goals are proofed as follows.

Based on the idealized *Log*_Req_, *SR* is applied to yield BAN logic proof 1 (*BP*_1_)

$$\begin{array}{c} BP_{1}:SGS_{\text{i}}⊲\{ñP,{\langle UID_{\text{i}}\rangle }_{ñP_{K}},{\langle R_{3}\rangle }_{h(UID_{\text{i}}\|{\text{SGS}}_{\text{MK}})},\\ {\langle SMID_{\text{i}}\rangle }_{h(UID_{\text{i}}|{|\text{R}}_{3}\text{)}},{\left(SMID_{\text{i}}\|R_{3}\right)}_{ñP_{K}},h(UID_{\text{i}}\|SGS_{\text{MK}})\} \end{array}$$

Using the *MMR* and *ISA*_6_ on *BP*_1_, we get *BP*_2_

$$\begin{array}{c} BP_{2}:SGS_{\text{i}}|\equiv U_{\text{i}}\sim \{ñP,{\langle UID_{\text{i}}\rangle }_{ñP_{K}},{\langle R_{3}\rangle }_{h(UID_{\text{i}}\|SGS_{\text{MK}})},\\ \;{\langle SMID_{\text{i}}\rangle }_{h(UID_{\text{i}}||R_{3}\text{)}},{\left(SMID_{\text{i}}\|R_{3}\right)}_{ñP_{K}},h(UID_{\text{i}}\|SGS_{\text{MK}})\} \end{array}$$

On the other hand, *FPR*, *ISA*_1_ and *NVR* are used in *BP*_2_ to yield *BP*_3_

$$\begin{array}{c} BP_{3}:SGS_{\text{i}}|\equiv U_{\text{i}}\equiv SGS_{\text{i}}⊲\{ñP,{\langle UID_{\text{i}}\rangle }_{ñP_{K}},{\langle R_{3}\rangle }_{h(UID_{\text{i}}\|SGS_{\text{MK}})},\\ {\langle SMID_{\text{j}}\rangle }_{h(UID_{\text{i}}||R_{3}\text{)}},{\left(SMID_{\text{j}}\|R_{3}\right)}_{ñP_{K}},h(UID_{\text{i}}\|SGS_{\text{MK}})\} \end{array}$$

Based on *BP*_3_, *ISA*_6_, *ISA*_12_ and *JR*, we obtain *BP*_4_

$$\begin{array}{c} BP_{4}:SGS_{\text{i}}|\equiv SGS_{\text{i}}⊲\{ñP,{\langle UID_{\text{i}}\rangle }_{ñP_{K}},{\langle R_{3}\rangle }_{h(UID_{\text{i}}\|SGS_{\text{MK}})},\\ {\langle SMID_{\text{i}}\rangle }_{h(UID_{\text{i}}||R_{3}\text{)}},{\left(SMID_{\text{i}}\|R_{3}\right)}_{ñP_{K}},h(UID_{\text{i}}\|SGS_{\text{MK}})\} \end{array}$$

Applying the *SKR* on *BP*_4_ results in *BP*_5_

$$BP_{5}:SGS_{\text{i}}|\equiv SGS_{\text{i}}\overset{{ɸ}_{\text{S}}}{↔}U_{\text{i}},$$

and hence **G-5** is achieved.

On the other hand, *ISA*_12_ and *NVR* are utilized in *BP*_5_ to obtain *BP*_6_

$$BP_{6}:SGS_{\text{i}}|\equiv U_{\text{i}}|\equiv SGS_{\text{i}}\overset{{ɸ}_{\text{S}}}{↔}U_{\text{i}},$$

effectively fulfilling **G-6**

However, applying *SR* to both idealized *Auth*_1_ and *Auth*_3_ results in *BP*_7_

$$\begin{array}{c} BP_{7}:SM_{\text{i}}⊲\{{\langle {\text{UID}}_{\text{i}}^{*}\rangle }_{SK_{S-SM},}{\langle R_{4}\rangle }_{h({\text{UID}}_{\text{i}}^{*}\|{\text{SG}}_{\text{S}-\text{SM}}^{*})},\\ {\langle R_{3}\rangle }_{R_{4}},{\left(UID_{\text{i}}\|SMID_{\text{i}}\right)}_{\left(R_{3},R_{4},{\text{SG}}_{\text{S}-\text{SM}}^{*}\right)}\} \end{array}$$

$$BP_{8}:U_{\text{i}}⊲\{{\langle R_{4}\rangle }_{h(UID_{\text{i}}\|{\text{SGS}}_{\text{MK}})},{\langle R_{5}^{*}\rangle }_{R_{3}^{*}},\;{\left({\text{UID}}_{\text{i}}^{*}\right)}_{\left(R_{4},\;R_{5}^{*},{ɸ}_{S}\right)}\}$$

Using *ISA*_9_ and the *MMR* on *BP*_7_ yields *BP*_9_

$$\begin{array}{c} BP_{9}:SM_{\text{i}}|\equiv SGS_{\text{i}}\sim \{{\langle {\text{UID}}_{\text{i}}^{*}\rangle }_{SK_{S-SM}},{\langle R_{4}\rangle }_{h({\text{UID}}_{\text{i}}^{*}\|SG_{\text{S}-\text{SM}}^{*})},\\ {\langle R_{3}\rangle }_{R_{4}},{\left(UID_{\text{i}}\|SMID_{\text{i}}\right)}_{\left(R_{3},R_{4},SG_{\text{S}-\text{SM}}^{*}\right)}\} \end{array}$$

On the other hand, applying *ISA*_4_ and the *MMR* on *BP*_8_ results in *BP*_10_

$$\begin{array}{c} BP_{10}:U_{i}|\equiv SGS_{\text{i}}\sim \{{\langle R_{4}\rangle }_{h(UID_{\text{i}}\|{\text{SGS}}_{\text{MK}})},\\ {\langle R_{5}^{*}\rangle }_{R_{3}^{*}},{\left(UID_{\text{i}}^{*}\right)}_{\left(R_{4},\;R_{5}^{*},{ɸ}_{S}\right)}\} \end{array}$$

Based on *FPR*, *NVR*, *ISA*_2_, *ISA*_14_ and *BP*_9_, we get *BP*_11_

$$\begin{array}{c} BP_{11}:SM_{\text{i}}|\equiv SGS_{\text{i}}|\equiv \{{\langle UID_{\text{i}}^{*}\rangle }_{SK_{S-SM}},{\langle R_{4}\rangle }_{h(UID_{\text{i}}^{*}\|SG_{\text{S}-\text{SM}}^{*})},\\ \;{\langle R_{3}\rangle }_{R_{4}},{\left(UID_{\text{i}}\|SMID_{\text{i}}\right)}_{\left(R_{3},R_{4},SG_{\text{S}-\text{SM}}^{*}\right)}\} \end{array}$$

However, according to *FPR*, *NVR*, *BP*_10_, *ISA*_2_ and *ISA*_11_, *BP*_12_ is obtained

$$\begin{array}{c} BP_{12}:U_{i}|\equiv SGS_{\text{i}}|\equiv \{{\langle R_{4}\rangle }_{h(UID_{\text{i}}\|SGS_{\text{MK}})},\\ \;{\langle R_{5}^{*}\rangle }_{R_{3}^{*}},{\left(UID_{\text{i}}^{*}\right)}_{\left(R_{4},\;R_{5}^{*},{ɸ}_{S}\right)}\} \end{array}$$

According to *JR*, *ISA*_11_ and *BP*_12_, we obtain *BP*_13_

$$\begin{array}{c} BP_{13}:U_{i}|\equiv \{{\langle R_{4}\rangle }_{h(UID_{\text{i}}\|SGS_{\text{MK}})},\\ \;{\langle R_{5}^{*}\rangle }_{R_{3}^{*}},{\left(UID_{\text{i}}^{*}\right)}_{\left(R_{4},\;R_{5}^{*},{ɸ}_{S}\right)}\} \end{array}$$

The application of the *SKR* on *BP*_13_ results in *BP*_14_

$$BP_{14}:SM_{\text{i}}|\equiv SM_{\text{i}}\overset{{ɸ}_{\text{M}}}{↔}SGS_{\text{i}},\;\text{hence}\;SM_{\text{i}}|\equiv \overset{{ɸ}_{\text{M}}}{↔}U_{\text{i}}.$$

Therefore, **G-1** is achieved.

Based on *BP*_13_ and *ISA*_14_, *SKR* is deployed to yield *BP*_15_

$$BP_{15}:SM_{\text{i}}|\equiv SGS_{\text{i}}|\equiv SM_{\text{i}}\overset{{ɸ}_{\text{M}}}{↔}SGS_{\text{i}},\;\text{and}\;\text{hence}\;SM_{\text{i}}|\equiv U_{\text{i}}|\equiv SM_{\text{i}}\overset{{ɸ}_{\text{M}}}{↔}U_{\text{i}}.$$

As such, **G-2** is realized.

Conversely, *SKR* is applied on *BP*_14_ to yield *BP*_16_

$$BP_{16}:U_{\text{i}}|\equiv U_{\text{i}}\overset{{ɸ}_{\text{U}}}{↔}SGS_{\text{i}},\;\text{and}\;\text{therefore}\;U_{\text{i}}|\equiv SM_{\text{i}}\overset{{ɸ}_{\text{U}}}{↔}U_{\text{i}}.$$

This effectively attains **G-3**.

Similarly, the application of *SKR* on *BP*_14_, *ISA*_5_ and *ISA*_11_ results in *BP*_17_

$$BP_{17}:U_{\text{i}}|\equiv SGS_{\text{i}}|\equiv U_{\text{i}}\overset{{ɸ}_{\text{U}}}{↔}SGS_{\text{i}},\;\text{hence}\;U_{\text{i}}|\equiv SM_{\text{i}}|\equiv SM_{\text{i}}\overset{{ɸ}_{\text{U}}}{↔}U_{\text{i}}.$$

This means that **G-4** is realized.

Applying *SR* idealized to *Auth*_2_ results in *BP*_18_

$$BP_{18}:SGS_{\text{i}}⊲\{{\langle R_{5}\rangle }_{SK_{S-SM}},{\left(R_{5}\right)}_{\left({ɸ}_{M,}SG_{\text{S}-\text{SM}}^{*}\right)}\}$$

Next, the *MMR* is utilized in *ISA*_7_ and *BP*_18_ to get *BP*_19_

$$BP_{19}:SGS_{\text{i}}|\equiv SM_{\text{i}}\sim \{{\langle R_{5}\rangle }_{SK_{S-SM}},{\left(R_{5}\right)}_{\left({ɸ}_{M,}SG_{\text{S}-\text{SM}}^{*}\right)}\}$$

Based on *ISA*_3_ and *BP*_19_, *FPR* and *NVR* are applied to get *BP*_20_

$$BP_{20}:SGS_{\text{i}}|\equiv SM_{\text{i}}|\equiv \{{\langle R_{5}\rangle }_{SK_{S-SM}},{\left(R_{5}\right)}_{\left({ɸ}_{M,}SG_{\text{S}-\text{SM}}^{*}\right)}\}$$

This is followed by the application of *JR* to *ISA*_7_, *ISA*_13_ and *BP*_20_ to obtain *BP*_21_

$$BP_{21}:SGS_{\text{i}}|\equiv \{{\langle R_{5}\rangle }_{SK_{S-SM}},{\left(R_{5}\right)}_{\left({ɸ}_{M,}SG_{\text{S}-\text{SM}}^{*}\right)}\}$$

According to *ISA*_8_, the usage of *SKR* in *BP*_21_ yields *BP*_22_

$$BP_{22}:SGS_{\text{i}}|\equiv SGS_{\text{i}}\overset{{ɸ}_{\text{S}}}{↔}SM_{\text{i}},$$

effectively realizing **G-7**.

Finally, SKR is applied to *ISA*_13_, *ISA*_15_ and *BP*_21_ to get *BP*_23_

$$BP_{23}:SGS_{\text{i}}|\equiv SM_{\text{i}}|\equiv SGS_{\text{i}}\overset{{ɸ}_{\text{S}}}{↔}SM_{\text{i}}.$$

This attains **G-8**.

The BAN logic proofs executed above confirms the attainment of mutual AKA procedures among the smart grid entities. In addition, it affirms the negotiation of session keys *ɸ*_S_ = *ɸ*_U_ = *ɸ*_M_ between the *U*_i_ and *SM*_i_ with the help of the *SGS*_i_.

### B. Informal security analysis

In this sub-section, our scheme is shown to be robust under all the assumptions of the Dolev-Yao (DY) and Canetti- Krawczyk (CK) threat models. To accomplish this, the following lemmas are devised and proofed.

*Lemma 1*: *Privileged insider and masquerading attacks are prevented*.

**Proof**: The assumption made here is that highly privileged entities such as the smart grid server administrator is interested in obtaining the registration information for some particular users. Afterwards, an attempt is made to impersonate this particular user *U*_i_. During *U*_i_ registration with the *SGS*_i_, registration request *Reg*_1_ = *{UID*_i_, *A*_1_, *Bio*_i_*}* is transmitted. Suppose that adversary *Å* wants to recover user password *PW*_i_ from *A*_1_, where *A*_1_ = *h* (*R*_2_||*PW*_i_). However, *PW*_i_ is encapsulated in random nonce *R*_2_ before being one-way hashed. Mathematically, it is impossible to reverse the one-way hash function. As such, the recovery of *PW*_i_ from *A*_1_ is not possible. Therefore, masquerading and privileged insider attacks are thwarted.

*Lemma 2*: *This protocol is robust against replay and de-synchronization attacks*

**Proof**: In this protocol, random parameters *R*_3_, *ñ*, *R*_4_ and *R*_5_ are incoporated in the exchanged messages. For instance, message *Log*_Req_, *Auth*_1_, *Auth*_2_ and *Auth*3 all incorporate random parameters. Here, *Log*_Req_ = {*A*_5_, *B*_2_, *B*_3_, *B*_4_, *B*_5_}, *Auth*_1_ = {*C*_1_, *C*_2_, *C*_3_, *C*_4_}, *Auth*_2_ = {*C*_5_, *D*_1_}, *Auth*_3_ = {*D*_2_, *D*_3_, *D*_4_}, *A*_5_ = *ñP*, *B*_2_ = *UID*_i_⊕*B*_1_, *B*_1_ = *ñP*_*K*_ = *ñPR*_1_, *B*_3_ = *A*_4_⊕*R*_3_, *B*_4_ = *h* (*UID*_i_||R_3_)⊕*SMID*_i_, *B*_5_ = *h* (*A*_4_||*SMID*_i_||*B*_1_||*R*_3_), *SMID*_i_* = *B*_4_⊕*h*(*UID*_i_*||*R*_3_*), *SK*_S-SM_* = *h* (*SMID*_i_*||*SGS*_MK_), *C*_1_ = *UID*_i_* ⊕ *SK*_S-SM_*, *C*_2_ = *R*_4_ ⊕ *h*(*UID*_i_*||*SK*_S-SM_*), *C*_3_ = *R*_4_ ⊕ *R*_3_*, *C*_4_ = *h* (*UID*_i_*||*SMID*_i_*||*SK*_S-SM_*||*R*_3_*||*R*_4_), *C*_5_ = *R*_5_ ⊕ *SK*_S-SM_, *D*_1_ = *h* (*SK*_S-SM_||*ɸ*_M_||*R*_5_), *D*_2_ = *A*_4_*⊕*R*_4_, *D*_3_ = *R*_3_*⊕*R*_5_* and *D*_4_ = *h* (*UID*_i_*||*ɸ*_S_||*R*_4_||*R*_5_*). Whereas *R*_3_ and *ñ* are generated by the user *U*_i_, *R*_4_ is generated at the *SGS*_i_. On the other hand, random nonce *R*_5_ is generated at the *SM*_i_. These random parameters ensure the freshness of the exchanged messages during a given communication session. However, in most schemes replay attacks prevention involve timestamps. Unfortunately, attackers can easily mount de-synchronization attacks riding on the deployed timestamps. Since the proposed protocol is devoid of timestamps, de-synchronization attacks are not possible.

*Lemma 3*: *Smart meter anonymity and untraceability are preserved*.

**Proof**: During the login and authentication phases, messages *Log*_Req_, *Auth*_1_, *Auth*_2_ and *Auth*_3_ are exchanged. Here, *Log*_Req_ = {*A*_5_, *B*_2_, *B*_3_, *B*_4_, *B*_5_}, *Auth*_1_ = {*C*_1_, *C*_2_, *C*_3_, *C*_4_}, *Auth*_2_ = {*C*_5_, *D*_1_}, *Auth*_3_ = {*D*_2_, *D*_3_, *D*_4_}, *C*_1_ = *UID*_i_*⊕*SK*_S-SM_*,*C*_2_ = *R*_4_⊕*h*(*UID*_i_*||*SK*_S-SM_*), *C*_3_ = *R*_4_⊕*R*_3_*, *C*_4_ = *h* (*UID*_i_*||*SMID*_i_*||*SK*_S-SM_*||*R*_3_*||*R*_4_), *C*_5_ = *R*_5_⊕*SK*_S-SM_, *D*_1_ = *h* (*SK*_S-SM_||*ɸ*_M_||*R*_5_), *D*_2_ = *A*_4_*⊕*R*_4_, *D*_3_ = *R*_3_*⊕*R*_5_*, *D*_4_ = *h* (*UID*_i_*||*ɸ*_S_||*R*_4_||*R*_5_*), *A*_5_ = *ñP*, *B*_2_ = *UID*_i_⊕*B*_1_, *B*_1_ = *ñP*_*K*_ = *ñPR*_1_, *B*_3_ = *A*_4_⊕*R*_3_, *B*_4_ = *h* (*UID*_i_||R_3_)⊕*SMID*_i_ and *B*_5_ = *h* (*A*_4_||*SMID*_i_||*B*_1_||*R*_3_). Clearly, none of these messages carry the plain-text smart meter identity *SMID*_i_*. Although parameter *C*_4_ contain *SMID*_i_*, it is encapsulated in other security parameters before being enciphered using one-way hashing function *h*(.). Suppose that an attacker *Å* wants to recover *SMID*_i_* from *Log*_Req_. However, this requires knowledge of the *SGS*_i_ secret key *R*_1_ and master key *SGS*_MK_. The inclusion of random nonces in the exchanged messages ensures that these messages are dynamically changed after each communication session. As such, it is cumbersome for *Å* to trace diverse sessions initiated by particular smart meters.

*Lemma 4*: *The entities mutually authenticate each other and negotiate session keys*.

**Proof**: In this scheme, the *SGS*_i_ is a trusted entity and serves to bridge the communication between *U*_i_ and *SM*_i_. The mutual authentication among these three communicating entities is both implicit and explicit. During the login procedures, *U*_i_ constructs and transmits login request message *Log*_Req_ = {*A*_5_, *B*_2_, *B*_3_, *B*_4_, *B*_5_} to the *SGS*_i_. Upon receiving this message, the *SGS*_i_ utilizes *R*_1_ to recover *UID*_i_* = *B*_2_⊕*B*_1_*. Here, *B*_1_* = *A*_5_*R*_1_, *B*_1_ = *ñP*_*K*_ = *ñPR*_1_ and *B*_2_ = *UID*_i_⊕*B*_1_. It then confirms if *UID*_i_* is in its database such that the login request is denied if it is not. To authenticate *U*_i_, the *SGS*_i_ re-computes *A*_4_*, *R*_3_*, *SMID*_i_* and *B*_5_*. Here, *A*_4_* = *h*(*UID*_i_*||*SGS*_MK_), *R*_3_* = *B*_3_⊕*A*_4_*, *SMID*_i_* = *B*_4_⊕*h*(*UID*_i_*||*R*_3_*) and *B*_5_* = *h* (*A*_4_*|| *SMID*_i_*||*B*_1_*||*R*_3_*). This is followed by the confirmation of whether *B*_5_* ≟ *B*_*5*_ such that the session is aborted if these parameters are dissimilar. On the other hand, upon receiving authentication message *Auth*_1_ from the *SGS*_i_, *SM*_i_ uses *SK*_S-SM_ to re-compute parameters *UID*_i_**, *R*_4_*, *R*_3_** and *C*_4_*. Here, *UID*_i_** = *C*_1_⊕*SK*_S-SM_, *R*_4_* = *h* (*UID*_i_**||*SK*_S-SM_)⊕ *C*_2_, *R*_3_** = *R*_4_*⊕*C*_3_ and *C*_4_* = *h* (*UID*_i_**||*SMID*_i_||*SK*_S-SM_||*R*_3_**||*R*_4_*). Thereafter, the *SGS*_i_ is authenticated by checking whether *C*_4_* ≟ *C*_*4*_ such that the session is rejected if this validation flops. Similarly, upon receiving authentication message *Auth*_2_ = {*C*_5_, *D*_1_} from the *SM*_i_, the *SGS*_i_ re-calculates parameter *R*_5_* using *SK*_S-SM_*. Next, it computes the session key *ɸ*_S_ = *h* (*UID*_i_*||*SMID*_i_*||*R*_3_*||*R*_4_||*R*_5_*) together with parameter *D*_1_* = *h* (*SK*_S-SM_*||*ɸ*_S_||*R*_5_*). This is followed by the confirmation of whether *D*_1_* ≟ *D*_*1*_. Alternatively, after receiving authentication message *Auth*_3_ from *SGS*_i_, the user *U*_i_ re-computes parameters *R*_4_** and *R*_5_** before deriving session key *ɸ*_U_ together with security parameter *D*_4_*. Finally, *U*_i_ authenticates the *SGS*_i_ by checking whether *D*_4_* ≟ *D*_*4*_. Clearly, all the three entities have successfully authenticated each other and derived session key for traffic enciphering.

*Lemma 5*: *User untraceability and anonymity are upheld*.

**Proof**: The goal of this security property is to conceal the user’s real identity from possible adversaries. This effectively helps foster high privacy in sensitive application domains such as in smart grids where captured consumption reports can aid attackers to discern the status of home occupancy. In our scheme, the user’s real identity is never sent in plain-text in any of the exchanged messages. The parameters *B*_2_ = *UID*_i_⊕*B*_1_ and *C*_1_ = *UID*_i_*⊕*SK*_S-SM_* encapsulate *UID*_i_ in login message *Log*_Req_ = {*A*_5_, *B*_2_, *B*_3_, *B*_4_, *B*_5_} and authentication message *Auth*_1_ = {*C*_1_, *C*_2_, *C*_3_, *C*_4_}. Using master key *R*_1_, the *SGS*_i_ can to re-compute *B*_1_* = *A*_5_*R*_1_ = *ñPR*_1_ and *UID*_i_* = *B*_2_⊕*B*_1_*. On the other hand, on receiving *Auth*_1_ from the *SGS*_i_, the *SM*_i_ utilizes secret key *SK*_S-SM_ to re-compute this identity as *UID*_i_** = *C*_1_⊕*SK*_S-SM_. Therefore, without knowledge of *R*_1_ and *SK*_S-SM_ adversary *Å* is unable to recover user identity from the exchanged messages. To uphold untraceability, *Å* should be unable to trace diverse sessions initiated by a particular user based on the captured exchanged messages. To accomplish this, random nonces *R*_3_ and *ñ* are generated by *U*_i_ for each of these communication sessions. As such, the login message *Log*_Req_ = {*A*_5_, *B*_2_, *B*_3_, *B*_4_, *B*_5_} is different for each session. Here, *A*_5_ = *ñP*,*B*_1_ = *ñP*_*K*_, *B*_2_ = *UID*_i_⊕*B*_1_, *B*_3_ = *A*_4_⊕*R*_3_, *B*_4_ = *h* (*UID*_i_||R_3_)⊕*SMID*_i_ and *B*_5_ = *h* (*A*_4_||*SMID*_i_||*B*_1_||*R*_3_).

*Lemma 6*: *This protocol protects against smart card loss attacks*.

**Proof**: The objective of this adversary is to deploy side-channeling techniques such as power analysis to discern the tokens buffered in the smart card. In the proposed protocol, the *U*_SC_ holds parameter set {*ē*, *f* (.), *ū*, *A*_*2*_, *A*_*3*_, *P*_*K*_, *R*_2_}. Here, *ē = h* (*RC*_i_), *ū = RC*_*i*_⊕*Bio*_*i*_, *A*_2_ = *h* (*UID*_*i*_||*A*_1_||*RC*_i_), *A*_1_ = *h* (*R*_2_||*PW*_i_) and *A*_3_ = *h* (*UID*_*i*_||*SGS*_MK_)⊕*h* (*h (R*_2_||*PW*_i_)||*RC*_*i*_). On the other hand, *RC*_i_ is the code-phrase chosen by the *SGS*_i_. Similarly, *R*_2_ is the random nonce generated by the user *U*_i_. The assumption made is that adversary *Å* has stolen the *U*_SC_ and is interested in recovering user identity *UID*_*i*_ and password *PW*_i_. To retrieve both *UID*_*i*_ and *PW*_i_ from *A*_2_, *Å* must be in possession of *RC*_i_ and be able to reverse the one-way hashing function *h*(.). Similarly, any successful recovery of *UID*_*i*_ and *PW*_i_ from *A*_3_ requires reversing *h*(.) and knowledge of *RC*_i_ and smart grid server master key *SGS*_MK_. Owing to unavailability of both *RC*_i_ and *SGS*_MK_ coupled with the difficulty of reversing *h*(.) implies the resilience of these attacks.

*Lemma 7*: *This scheme is resilience against forgery attacks*.

**Proof**: The goal of this attack is to deploy intercepted messages exchanged over public channels to discern security parameters belonging to the communicating entities. Thereafter, attempts are made to falsify messages such that they appear to emanate from valid entities. Suppose that an adversary *Å* is interested in falsifying login request message *Log*_Req_ = {*A*_5_, *B*_2_, *B*_3_, *B*_4_, *B*_5_}. To achieve this, *Å* needs to recover user identity *UID*_i_ and parameter *A*_4_ = *A*_3_⊕*h* (*h(R*_2_||*PW*_i_||*RC*_i_*). However, based on *Lemma 5*, *UID*_i_ cannot be recovered from the publicly exchanged messages. On the other hand, the adversary needs password *PW*_i_, *R*_2_ and *RC*_i_* to correctly re-compute *A*_4_. Suppose that *Å* has access to the user’s smart card *U*_SC_. However, based on *Lemma 6*, the adversary is unable to falsify the user using the security tokens in the *U*_SC_. On the other hand, any successful falsification of the *SGS*_i_ messages calls for the knowledge of private key *R*_1_ and master key *SGS*_MK_. Since these parameters cannot be eavesdropped over the public channel, this attack fails. Similarly, *Å* cannot falsify *SM*_i_ messages devoid of secret key *SK*_S-SM_, where *SK*_S-SM_* = *h* (*SMID*_i_*||*SGS*_MK_).

*Lemma 8*: *KSSTI attack is prevented in this protocol*.

**Proof**: To prevent this attack, all the three communicating entities negotiate session keys *ɸ*_U_ = *ɸ*_S_ = *ɸ*_M_ that are derived using random nonces *R*_3_, *R*_4_ and *R*_5_ as well identities *UID*_*i*_ and *SMID*_i_. Here, *ɸ*_M_ = *h* (*UID*_i_**||*SMID*_i_||*R*_3_**||*R*_4_*||*R*_5_), *ɸ*_S_ = *h* (*UID*_i_*||*SMID*_i_*||*R*_3_*||*R*_4_||*R*_5_*) and *ɸ*_U_ = *h* (*UID*_i_||*SMID*_i_||*R*_3_||*R*_4_**||*R*_5_**). Based on *Lemma 3* and *Lemma 5* adversary *Å* is unable to recover *UID*_*i*_ and *SMID*_i_ from the exchanged messages *Auth*_1_, *Auth*_2_ and *Auth*_3_. Suppose that *Å* has access to the random nonces *R*_3_, *R*_4_ and *R*_5_. The next objective is to derive the session keys using these nonces. However, devoid of the knowledge of user identity *UID*_*i*_ and smart meter identity *SMID*_i_, this derivation flops.

*Lemma 9*: *This scheme is robust against guessing attacks*.

**Proof**: The thwarting of invalid logins is critical for prevention of unnecessary computation and communication overheads. To this end, fuzzy commitment technique is deployed to validate the biometric information imprinted by *U*_i_. In the login phase, the user has to insert *U*_SC_ into its reader and imprint biometric *Bio*_i_*on some special device. Afterwards, the smart card derives parameter $RC_{\text{i}}*=f(ū⊕Bio_{\text{i}}^{*})=f(RC_{\text{i}}⊕(Bio_{i}⊕Bio_{\text{i}}^{*})$. To validate the imprinted *Bio*_i_*, it checks whether *h* (*RC*_i_*) ≟ *ē = h* (*RC*_i_). It is only after successful biometric verification that *U*_i_ can proceed to input *UID*_i_ and *PW*_i_. Essentially, biometric, identity and password validation must be successful before *U*_i_ can be allowed access to the smart grid systems. As such, it is difficult for the adversary *Å* to correctly guess all these three security parameters so as to gain access.

*Lemma 10*: *This protocol can withstand MitM attacks*

**Proof**: The objective of adversary here is to intercept the transmitted messages, change them before forwarding them to the unsuspicious destination terminals. In the proposed protocol, 4 messages are exchanged during the login and AKA procedures. The four messages include *Log*_Req_ = {*A*_5_, *B*_2_, *B*_3_, *B*_4_, *B*_5_}, *Auth*_1_ = {*C*_1_, *C*_2_, *C*_3_, *C*_4_}, *Auth*_2_ = {*C*_5_, *D*_1_} and *Auth*_3_ = {*D*_2_, *D*_3_, *D*_4_}. Here, *A*_5_ = *ñP*, *B*_2_ = *UID*_i_⊕*B*_1_, *B*_1_ = *ñP*_*K*_, *B*_3_ = *A*_4_⊕*R*_3_, *B*_4_ = *h* (*UID*_i_||R_3_)⊕*SMID*_i_, *B*_5_ = *h* (*A*_4_||*SMID*_i_||*B*_1_||*R*_3_), *C*_1_ = *UID*_i_*⊕*SK*_S-SM_*,*C*_2_ = *R*_4_⊕*h*(*UID*_i_*||*SK*_S-SM_*), *C*_3_ = *R*_4_⊕*R*_3_*, *C*_4_ = *h* (*UID*_i_*||*SMID*_i_*||*SK*_S-SM_*||*R*_3_*||*R*_4_), *C*_5_ = *R*_5_⊕*SK*_S-SM_, *ɸ*_M_ = *h* (*UID*_i_**||*SMID*_i_||*R*_3_**||*R*_4_*||*R*_5_), *D*_1_ = *h* (*SK*_S-SM_||*ɸ*_M_||*R*_5_), *D*_2_ = *A*_4_*⊕*R*_4_, *D*_3_ = *R*_3_*⊕*R*_5_* and *D*_4_ = *h* (*UID*_i_*||*ɸ*_S_||*R*_4_||*R*_5_*). Suppose that an attacker *Å* wants to construct bogus messages *Log*_Req_^Å^, *Auth*_1_^Å^, *Auth*_2_^Å^ and *Auth*_3_^Å^. However, this requires correct guessing of the random nonces *R*_3_, *ñ*, *R*_4_ and *R*_5_. In addition *Å* needs to have knowledge of user identity *UID*_i_, smart meter identity *SMID*_i_, secret key *SK*_S-SM_ as well as session key *ɸ*_M_. Based on *Lemma 3* and *Lemma 5*, *UID*_i_ and *SMID*_i_ cannot be eavesdropped by *Å*. By *Lemma 2*, nonces *R*_3_, *ñ*, *R*_4_ and *R*_5_ are stochastic and hence cannot be correctly derived by the adversary. On the other hand, *Lemma 8* illustrates the difficulty of deriving session keys *ɸ*_M_, *ɸ*_U_ and *ɸ*_S_. All these coupled with the challenges of obtaining secret key *SK*_S-SM_ proofs that our protocol can withstand these attacks.

*Lemma 11*: *This protocol is robust against physical capture attacks*

**Proof**: Suppose that an adversary *Å* has gained physical access to the smart meter *SM*_i_. The next goal is to retrieve the parameters stored in *SM*_i_ so as to derive the session keyn *ɸ*_M_ = *h* (*UID*_i_**||*SMID*_i_||*R*_3_**||*R*_4_*||*R*_5_). To derive any valid *ɸ*_M_, *Å* needs to compute *UID*_i_** = *C*_1_⊕*SK*_S-SM_ and random nonces *R*_3_** = *R*_4_*⊕*C*_3_, *R*_4_* = *h* (*UID*_i_**||*SK*_S-SM_)⊕ *C*_2_ and *R*_5_. Here, *C*_1_ = *UID*_i_*⊕*SK*_S-SM_*, *C*_2_ = *R*_4_⊕*h*(*UID*_i_*||*SK*_S-SM_*), *C*_3_ = *R*_4_⊕*R*_3_*, *R*_3_* = *B*_3_⊕*A*_4_*, *B*_3_ = *A*_4_⊕*R*_3_, *A*_4_ = *A*_3_⊕*h* (*h(R*_2_||*PW*_i_||*RC*_i_*), *A*_3_ = *h* (*UID*_*i*_||*SGS*_MK_)⊕*h* (*A*_*1*_||*RC*_*i*_), *A*_1_ = *h* (*R*_2_||*PW*_i_) and random code-phrase *RC*_*i*_ ∈ *CS*. During the registration phase, parameter set {*SMID*_i_, *SK*_S-SM_} is privately stored in *SM*_i_’s memory. Although the attacker has access to *SMID*_i_ and *SK*_S-SM_, it is computationally infeasible to simultaneously and correctly guess random nonces deployed in *ɸ*_M_. In addition, *Å* requires user password *PW*_i_. However, based on *Lemma 1*, the adversary is unable to obtain this password and hence this protocol is resilient against smart meter physical capture attacks.

*Lemma 12*: *This scheme can withstand spoofing attacks*

**Proof**: The aim of the attacker here is to attempt to present security parameters belonging to other legitimate entities for the authentication process. During the login and AKA procedures, the *SGS*_i_ authenticates *U*_i_ by checking whether *B*_5_* ≟ *B*_5_. On the other hand, the *SM*_i_ authenticates *SGS*_i_ by confirming if *C*_4_* ≟ *C*_4_. Similarly, the *SGS*_i_ validates *SM*_i_ by checking if *D*_1_* ≟ *D*_1_. Finally, the *U*_i_ authenticates *SGS*_i_ through the confirmation of whether *D*_4_* ≟ *D*_4_. Here, *B*_5_ = *h* (*A*_4_||*SMID*_i_||*B*_1_||*R*_3_), *B*_5_* = *h* (*A*_4_*||*SMID*_i_*||*B*_1_*||*R*_3_*), *C*_4_ = *h* (*UID*_i_*||*SMID*_i_*||*SK*_S-SM_*||*R*_3_*||*R*_4_), *C*_4_* = *h* (*UID*_i_**||*SMID*_i_||*SK*_S-SM_||*R*_3_**||*R*_4_*), *D*_1_ = *h* (*SK*_S-SM_||*ɸ*_M_||*R*_5_), *D*_1_* = *h* (*SK*_S-SM_*||*ɸ*_S_||*R*_5_*), *D*_4_ = *h* (*UID*_i_*||*ɸ*_S_||*R*_4_||*R*_5_*) and *D*_4_* = *h* (*UID*_i_||*ɸ*_U_||*R*_4_**||*R*_5_**). It is evident that devoid of correct *SMID*_i_, *UID*_i_, *SK*_S-SM_*, *ɸ*_M_, *ɸ*_U_ and random nonces, authentication using spoofed parameters will fail.

## 6. Performance evaluation

Computation, storage, supported security features and communication complexities are the most predominant parameters for appraising the performance of security schemes. As such, these four metrics are utilized in this section to appraise the developed scheme.

### A. Computation complexity

In most of the authentication and key negotiation process, executions times for map to point hash (*T*_MH_), symmetric encryption or decryption (*T*_ED_), one-way hashing operations (*T*_H_), Hash-based Message Authentication Code (*T*_HMAC_), ECC point multiplications (*T*_EM_), modular exponentiation (*T*_E_), ECC point addition (*T*_EA_), pseudo-random function (*T*_PF_) and bilinear operation (*T*_BP_) are considered. Using the values in [25, 55, 58], Table 5 gives the execution times of these cryptographic primitives.

**Table 5:** Cryptographic execution times.

Operation	Execution time (ms)
Symmetric encryption or decryption, *T*_ED_	0.0046
One-way hashing operations, *T*_H_	0.0023
ECC point multiplications, *T*_EM_	2.226
Exponential operation. *T*_E_	3.8500
ECC point addition,*T*_EA_	0.0288
Bilinear operation, *T*_BP_	5.8110
Pseudo-random function, *T*_PF_	0.5390
Map to point hash, *T*_MH_	12.4180
Hash-based Message Authentication Code, *T*_HMAC_	6.176

In the process of executing login and AKA procedures, the *U*_i_ carries out 2 ECC point multiplications (*T*_EM_) and 8 one-way hashing operations (*T*_H_). On the other hand, the *SGS*_i_ carries out 1 *T*_EM_ operation and 9 *T*_H_ operations. However, the *SM*_i_ executes only 4*T*_H_ operations. As such, the execution time at the *U*_i_ is 4.4704 ms while this value is 2.2467 ms at the *SGS*_i_. On the other hand, the computation complexity at the *SM*_i_ is only 0.0092 ms. Therefore, the total computation cost is 6.7263 ms. However, considering only the *SM*_i_ and *SGS*_i_ residing on the utility control, the overall computation complexity is 2.2559 ms. Table 6 gives the comparison of the obtained computation cost against other state of the art protocols.

**Table 6:** Computation complexity comparisons.

Scheme	Smart meter + Utility control	Computation complexity (ms)
Mahmood et al. [25]	4*T*_EM_ + 3*T*_BP_ + 2*T*_E_ + 7 *T*_H_	34.0531
Mohammadali et al. [37]	5*T*_EM_ + 7 *T*_H_	11.1461
Kumar et al. [43]	4*T*_EM_ + 12 *T*_H_	8.9316
Nyangaresi et al. [53]	2*T*_EM_ + 3*T*_H_	4.4589
Kumar et al. [54]	5*T*_EM_ + 4*T*_HMAC_	17.3060
Challa et al. [56]	2*T*_EM_ + 20 *T*_H_	4.4980
Chaudhry et al. [57]	5*T*_EM_ + 2*T*_ED_ + 18 *T*_H_	11.1806
Mahmood et al. [61]	5*T*_EM_ + 5 *T*_H_ + 1 *T*_EA_	11.1703
Taqi et al. [62]	7 *T*_H_ + 4 *T*_ED_ + 6*T*_EM_	13.3905
Xia et al. [63]	37*T*_EM_	82.362
Proposed	13*T*_H_ + 1*T*_EM_	2.2559

**Fig 4:**
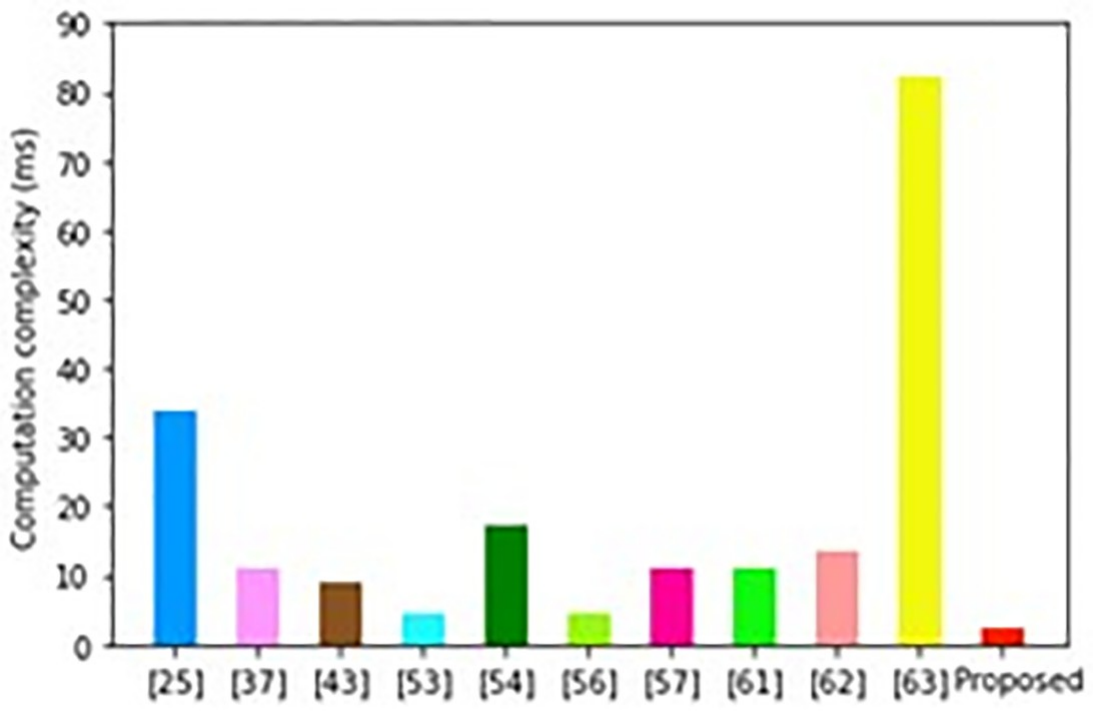
Computation complexity comparisons.

As illustrated in Fig 4, the scheme in [63] incurs the highest computation costs while the proposed protocol has the least computation overheads. Although the scheme in [56] has the second lowest computation complexity, it cannot provide authentication between two entities of the smart grid.

In addition, its design fails to consider forgery, smart card loss, guessing and de-synchronization attacks. Further, it cannot provide both user untraceability and anonymity.

### B. Communication complexity

During the AKA phase, messages, *Auth*_1_, *Auth*_2_ and *Auth*_3_ are exchanged. Here, *Auth*_1_ = {*C*_1_, *C*_2_, *C*_3_, *C*_4_}, *Auth*_2_ = {*C*_5_, *D*_1_}, *Auth*_3_ = {*D*_2_, *D*_3_, *D*_4_}, *C*_1_ = *UID*_i_*⊕*SK*_S-SM_*,*C*_2_ = *R*_4_⊕*h*(*UID*_i_*||*SK*_S-SM_*), *C*_3_ = *R*_4_⊕*R*_3_*, *C*_4_ = *h* (*UID*_i_*||*SMID*_i_*||*SK*_S-SM_*||*R*_3_*||*R*_4_), *C*_5_ = *R*_5_⊕*SK*_S-SM_, *D*_1_ = *h* (*SK*_S-SM_||*ɸ*_M_||*R*_5_), *D*_2_ = *A*_4_*⊕*R*_4_, *D*_3_ = *R*_3_*⊕*R*_5_* and *D*_4_ = *h* (*UID*_i_*||*ɸ*_S_||*R*_4_||*R*_5_*). Based on the values in [58, 71], SHA-1, ECC points, random nonce, identities and public or private keys are 160 bits, 320 bits, 160 bits, 64 bits and 160 bits respectively. As such, the length of *Auth*_1_, *Auth*_2_ and *Auth*_3_ are 640 bits, 320 bits and 480 bits respectively. Therefore, the cumulative bandwidth requirement is 1440 bits. The data of our experiments based on [72] data set. Table 7 presents the communication complexities comparisons with other schemes.

**Table 7:** Communication complexity comparisons.

Scheme	Communication complexity (bits)
Mahmood et al. [25]	1340
Mohammadali et al. [37]	1984
Kumar et al. [43]	1376
Nyangaresi et al. [53]	832
Kumar et al. [54]	2032
Challa et al. [56]	1536
Chaudhry et al. [57]	2080
Mahmood et al. [61]	2384
Taqi et al. [62]	1984
Xia et al. [63]	2816
Proposed	1440

**Fig 5:**
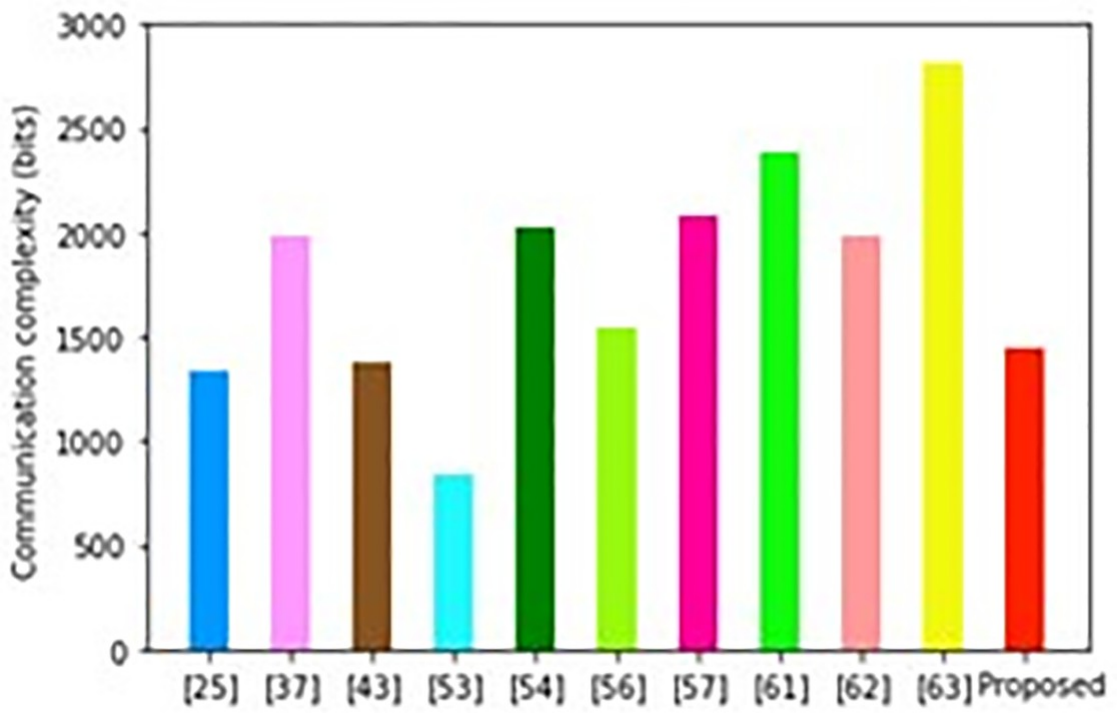
Communication complexity comparisons.

As illustrated in Fig 5, the scheme developed in [63] incurs the highest communication overheads while the scheme in [53] requires the least. Conversely, our scheme incurs the fourth lowest communication costs after the schemes in [25, 43, 53] respectively. However, each of these security approaches has some setbacks that render them unsuitable for deployment in smart grid environment. For instance, the scheme in [53] does not consider replay, privileged insider and masquerading and attacks in its design. On the other hand, the protocol in [25] cannot withstand ephemeral secret leakage and impersonation attacks.

Similarly, the approach in [43] can support only one smart meter and hence has scalability issues. In addition, its architecture does not consider smart card loss, forgery, mutual authentication, guessing, de-synchronization, user untraceability and anonymity.

### C. Space complexities

To derive the space complexity of our scheme, we consider the length of the parameters that have to be stored in the smart meter devices. During the registration phase, security parameter set {*SMID*_i_, *SK*_S-SM_} is privately stored in the memory of the *SM*_i_. Therefore, using the values in [58, 71], the space complexity at the *SM*_i_ and *U*_i_ is 320 bits. Table 8 offers the space comparisons with other related protocols.

**Table 8:** Space complexity comparisons.

Scheme	Space complexity (bits)
Mahmood et al. [25]	2048
Mohammadali et al. [37]	800
Kumar et al. [43]	480
Nyangaresi et al. [53]	160
Kumar et al. [54]	1280
Challa et al. [56]	640
Chaudhry et al. [57]	640
Mahmood et al. [61]	480
Taqi et al. [62]	544
Xia et al. [63]	1280
Proposed	320

**Fig 6:**
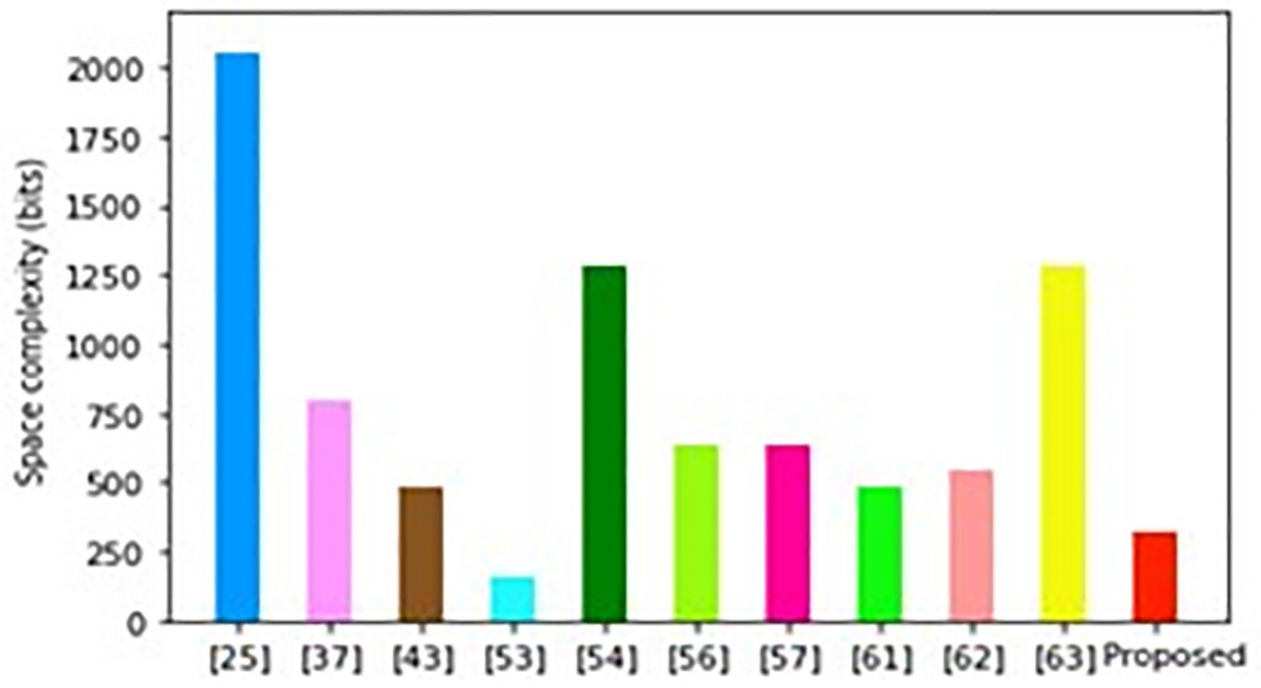
Space complexity comparisons.

As illustrated in Fig 6, the scheme in [25], incurs the highest space complexity while the approach in [53] exhibits the lowest. Conversely, the proposed protocol requires the second lowest space overheads of only 320 bits. However, it has already been noted that the protocol in [53] does not consider replay, masquerading and privileged insider attacks in its design. Evidently, all the schemes that perform slightly better than the proposed protocol have numerous performance, security and performance challenges. For example, the protocol in [56] has the lowest computation complexity but relatively higher communication and storage overheads. On the other hand, the technique in [53] incurs the least storage and communication costs but has relatively higher computation costs.

### D. Security features

To effectively appraise the developed protocol, its security goals and features are compared with those ones offered by other related state of the art protocols. Table 9 gives a summary of this comparison. It is evident from Table 9 that only our protocol that provides all the thirteen security characteristics. This is followed by the scheme in [54] which offers only 8 features. Next are the protocols in [25, 37, 56, 61, 62] which provide only 7 features each. The schemes in [43, 57] follow with 6 features each, while the approaches in [53, 63] offer only 3 features each. Therefore, although our scheme incurs slightly higher storage and communication costs, it is the most secure. Conversely, the scheme in [53] exhibits least communication and storage costs but is the most insecure among all the protocols. Consequently, the proposed protocol offers the best trade-off between performance and security. Therefore, it is the most ideal for deployment in high sensitive and resource-limited smart grid environment.

**Table 9:** Security features comparisons.

Scheme	F_1_	F_2_	F_3_	F_4_	F_5_	F_6_	F_7_	F_8_	F_9_	F_10_	F_11_	F_12_	F_13_
Mahmood et al. [25]	√	√	√	√	-	-	-	√	-	√	-	√	-
Challa et al. [56]	√	√	√	√	-	-	-	√	-	√	-	√	-
Nyangaresi et al. [53]	-	-	√	√	-	-	√	-	-	-	-	-	-
Kumar et al. [43]	√	√	√	-	-	-	-	√	-	√	-	√	-
Mohammadali et al. [37]	-	√	×	√	-	√	√	√	-	√	-	√	-
Chaudhry et al. [57]	√	√	√	-	-	-	-	√	-	√	-	√	-
Kumar et al. [54]	-	√	√	√	-	√	√	√	-	√	-	√	-
Mahmood et al. [61]	-	√	×	√	-	√	√	√	-	√	-	√	-
Taqi et al. [62]	√	√	√	-	√	√	-	-	√	√	-	-	×
Xia et al. [63]	√	√	-	-	-	-	-	-	-	√	-	-	-
Proposed	√	√	√	√	√	√	√	√	√	√	√	√	√

F_1_ -privileged insider and masquerading; F_2_- Replay; F_3_—smart meter anonymity and untraceability; F_4_- mutual authentication; F_5_—user untraceability and anonymity; F_6_—smart card loss; F_7_ -Forgery; F_8_—KSSTI; F_9_—Guessing; F_10_—man-in-the-middle; F_11_—physical capture; F_12_ -Spoofing; F_13_ -de-synchronization;√—provides security property; ×—Does not provide security property;—lacks security property

## 7. Conclusion

The smart grids offer efficiency in the management of demand responses in power systems. However, the exchange of consumption data over the open public channels implies that these messages are susceptible to a myriad of privacy and security violations. As such, previous researches have seen the introduction of numerous smart grid protection schemes. Unfortunately, many vulnerabilities have been discovered in these protocols that can be exploited to compromise smart grids. In addition, some of the current security solutions incur extensive storage, communication and computation complexities that limit their deployment in resource-constrained smart grid devices. To remedy some of these challenges, an efficient and provably secure protocol is introduced in this paper. The semantic security analysis has demonstrated its robustness under all the assumptions in the Dolev-Yao and Canetti- Krawczyk threat models. In addition, formal security analysis using the BAN logic has shown the attainment of strong mutual authentication and establishment of session keys among the smart grid entities. In terms of performance, it is shown to incur the lowest execution time, and relatively lower communication and space complexities compared with other related protocols. It is therefore applicable in resource-constrained smart grid environment where sensitive and private messages are exchanged. Future work in this domain will involve the analysis of this scheme using additional metrics that were not incorporated in the current work.

## Supporting information

S1 Data (DOCX)
