# Supplementary material for: Low complexity smart grid security protocol based on elliptic curve cryptography, biometrics and hamming distance
Source: PLoS One. 2024 Jan 23;19(1):e0296781. doi: 10.1371/journal.pone.0296781 (PMC10805298; doi:10.1371/journal.pone.0296781)
Supplement: S1 Data — (DOCX) [file pone.0296781.s001.docx]

| **Variable** | **Value** |
| --- | --- |
| $R_{1}$ | 8996cf56d8c9d6528416dbf7ff6cd29bf19aa8ecae07ea08a4b6d507bea565ad |
| *P*_K_ | c5ca4f817be17bab5fecd30faa766521196bac8ae8af4afb63a36cf8208d378c |
| *SMID*_i_ | 38a8f2a9346b8dfc |
| *SK*_S-SM_ | 0e98da609ab75ea6b11cbb16996bac19da517348 |
| *UID*_i_ | 38a8f2a9346b8dfc |
| *PW*_i_ | ff93b46f14b4aa18 |
| *R*_2_ | d653b016db1da8f5 |
| *A*_1_ | 691c532d787a69a0a61317c3a5086597f205f4e6 |
| ${RC}_{i}$ | d5eb20bf9be7480cc159e81cba5eeb380fb904026b87c50b71e8b5964ccf8ff9 |
| *ē* | a5f1750524c06203522a255b8cb032031c878154 |
| *ū* | 1100100001101010110010101100010001100100011000001100010011001100011100101100010011001010011011100110100001110000011000001100011011000110011000100110101001110010110010100111000001100010110001100989940743724779682128803203620825211889600543572171966413687543142113766497079974320309298101445788348850843888202243341729745657731642291331926371188709409783479570388130738266289149857662857280033939799020740254686538380780607115082426775770357510444178260755252908458508028591798249950900244390840087313151490242168 |
| *RC*_i_^*^ | d5eb20bf9be7480cc159e81cba5eeb380fb904026b87c50b71e8b5964ccf8ff9 |
| *A*_2_ | 543f71b47672010ada8d86618c82b8e3865e425f |
| *A*_3_ | 1450539270432371805443573321507644415812840360872909015661825681428910785723419459185028743258210320620349732963699967980532109602462703419436110657409098536373477562084403838365340537296987171926360744333048382612218975100987149689035014176903941859995181006608445187304162042340618842504969627937170402476177931 |
| *A_2_^*^* | 543f71b47672010ada8d86618c82b8e3865e425f |
| *R*_3_ | 21bbd75cf6530bd |
| *ñ* | 1b16b1df538ba12dc3f97edbb85caa7050d46c148134290feba80f8236c83db9 |
| *A*_4_ | 1409853016361484573314425292602425827578525404344123157015549424901873790267748389205559394278597630064467951528485498221535818729411988165203624404007673970059715671558079271080853122693836920821331630217836703367518130838934084592548277960182597340153789516372585036983800227218624833991424840750771115976225861630 |
| *A*_5_ | ff65a1e4f3aff0ca818e060793f661f50b4ffa6101272881046d6738ec392d62 |
| *B*_1_ | f8f749f566ce9e59fd75c9097c74b18e051b09dc17fcbe8559a10b9a1a576231 |
| *B*_2_ | 1100110001110000110011000110111001101000011100101100110001101010011011000110110011000110110010100111001011001010011010100111001011001100110010000110111001101010110001100111001001100000011100100110111011000110011011100110100011000100011000100111000011001010011000000110101001100010110001000110000001110010110010001100011001100010011011101100110011000110110001001100101001110000011010100364056466944287868507286590940011944091032545137736691000342002731907504508509975759551412295484991810311231303646277734255145 |
| *B*_3_ | 110000001101000011010100110011011000110110010100111000001101000011001000110110001101100011100100111001001110000011000001100010011000100011011100110000001101110011000001100100001110010011011100008076255852993139644687651889496755467087717995788786499020354014962767390938068065965234040186603558710901095717133091883600 |
| *B*_4_ | 110011011001010110010000110100001101100110011000110111001110000011010100110111001100110011010001100010011001010011011000111001011001000110000101100010001100100011100100110100001100110110001101207289600570904420359745420487881370444819965665975235287537813262130720625939347186346962372049836966500435663940359044557427 |
| *B*_5_ | ba6a3698e281cf61e46a24cbfa89e8f69361cf55 |
| *B*_1_^*^ | f8f749f566ce9e59fd75c9097c74b18e051b09dc17fcbe8559a10b9a1a576231 |
| *UID*_i_^*^ | 38a8f2a9346b8dfc |
| *A*_4_^*^ | 4d7b5df432ea603e8ac405a9f9f9fa6a080663cf |
| *R*_3_^*^ | 21bbd75cf6530bd |
| *SMID*_i_^*^ | 38a8f2a9346b8dfc |
| *B*_5_^*^ | ba6a3698e281cf61e46a24cbfa89e8f69361cf55 |
| *R*_4_ | ec7e008b5f4d05c4 |
| *SK*_S-SM_^*^ | 0e98da609ab75ea6b11cbb16996bac19da517348 |
| *C*_1_ | 110000011001010011100100111000011001000110000100110110001100000011100101100001011000100011011100110101011001010110000100110110011000100011000100110001011000110110001001100010001100010011011000534131841518480986163199585614132558553298865217399088770228764121759722345610579355736654357948996050473983737415882345809664 |
| *C*_2_ | 110111001101100011100001100110001100100011011100110000011001000110001101100001011001100011001000110111001100000011011101100110001110000110010100110000001100010011000101100110011001010011011101040870675192173501287305096302766911794614104895068605629542800170429399822925337301198414879166968163197149326676299970518693 |
| *C*_3_ | 1040705372113741508586802967943399731315840504655295870697453595421214145973752613396521065569366792053565148356417685706000976 |
| *C*_4_ | 61b273cb81267f8c329f62e4e661033952b2fa2b |
| *UID*_i_^**^ | 38a8f2a9346b8dfc |
| *R*_4_^*^ | ec7e008b5f4d05c4 |
| *R*_3_^**^ | 21bbd75cf6530bd |
| *C*_4_^*^ | 61b273cb81267f8c329f62e4e661033952b2fa2b |
| *R*_5_ | ec7e008b5f4d05c4 |
| *C*_5_ | 110000011001010011100100111000011001000110000100110110001100000011100101100001011000100011011100110101011001010110000100110110011000100011000100110001011000110110001001100010001100010011011000534138254325757911169290448333254508357869853560549017049372576949435139603313209109281414607332887624750802261553122095955084 |
| *ɸ*_M_ | f613925931cf6608320866056b07bf8933037d8f |
| *D*_1_ | 8957233b70eb19f2fcae73a4809a2892f6bc0057 |
| *R*_5_^*^ | ec7e008b5f4d05c4 |
| *ɸ*_S_ | f613925931cf6608320866056b07bf8933037d8f |
| *D*_1_^*^ | 8957233b70eb19f2fcae73a4809a2892f6bc0057 |
| *D*_2_ | 110000001101000011010100110011011000110110010100111000001101000011001000110110001101100011100100111001001110000011000001100010011000100011011100110000001101110011000001100100001110010011011099010051450964265742079589137699731194992864903261912928114341036078973839070394598257930246887590116111881251224439852217663488 |
| *D*_3_ | 1040705372113741508586802967943399731315840504655295870697453595421214145973752613396521065569366792053565148356417685706000976 |
| *D*_4_ | d5cb5bf7f1461216a61c8c0a931adae53f054b8e |
| *R*_4_^**^ | ec7e008b5f4d05c4 |
| *R*_5_^**^ | ec7e008b5f4d05c4 |
| *ɸ*_U_ | f613925931cf6608320866056b07bf8933037d8f |
| *D*_4_^*^ | d5cb5bf7f1461216a61c8c0a931adae53f054b8e |
| *PW*_i_^New^ | 098e3152e3e8cea6 |
| *A*_2_^New^ | 9d4f27984ad55d1ca0010e08648f35993437e653 |
| *A*_3_^New^ | 1100001011001000011000000110100001101010011100000111000001100010110011000110101001101110110011001100011001110000011000101100101001110000011100000111001001110010110011000110110011000010110000101100010011000100011100000110111001100000011100000110111001100100011001001100001011001000110010100110111011000010110010101100011 |
